# Associations of the *MTHFR* rs1801133 polymorphism with coronary artery disease and lipid levels: a systematic review and updated meta-analysis

**DOI:** 10.1186/s12944-018-0837-y

**Published:** 2018-08-17

**Authors:** Zhi Luo, Zhan Lu, Irfan Muhammad, Yun Chen, Qiuhong Chen, Jiaojiao Zhang, Yongyan Song

**Affiliations:** 1 Department of Cardiology, Affiliated Hospital of North Sichuan Medical College, Nanchong, 637000 People’s Republic of China; 2 School of Clinical Medicine, North Sichuan Medical College, Nanchong, 637000 People’s Republic of China; 3 Department of Medical Biochemistry, School of Preclinical Medicine, North Sichuan Medical College, Nanchong, 637000 People’s Republic of China

**Keywords:** 5,10-Methylenetetrahydrofolate reductase, Polymorphism, rs1801133, Coronary artery disease, Lipid

## Abstract

**Background:**

The associations of the 5,10-methylenetetrahydrofolate reductase gene (*MTHFR*) rs1801133 polymorphism with coronary artery disease (CAD) and plasma lipid levels have been widely investigated, but the results were inconsistent and inconclusive. This meta-analysis aimed to clarify the relationships of the rs1801133 polymorphism with CAD and plasma lipid levels.

**Methods:**

By searching in PubMed, Google Scholar, Web of Science, Cochrane Library, Wanfang, VIP and CNKI databases, 123 studies (87,020 subjects) and 65 studies (85,554 subjects) were identified for the CAD association analysis and the lipid association analysis, respectively. Odds ratio (OR) and standardized mean difference (SMD) were used to determine the effects of the rs1801133 polymorphism on CAD risk and lipid levels, respectively.

**Results:**

The variant T allele of the rs1801133 polymorphism was associated with increased risk of CAD under allelic model [OR = 1.11, 95% confidence interval (CI) = 1.06–1.17, *P* < 0.01], additive model (OR = 1.25, 95% CI = 1.14–1.37, *P* < 0.01), dominant model (OR = 1.11, 95% CI = 1.04–1.17, *P* < 0.01), and recessive model (OR = 1.22, 95% CI = 1.12–1.32, *P* < 0.01). The T carriers had higher levels of total cholesterol (TC) (SMD = 0.04, 95% CI = 0.01–0.07, *P* = 0.02) and low-density lipoprotein cholesterol (LDL-C) (SMD = 0.07, 95% CI = 0.01–0.12, *P* = 0.01) than the non-carriers.

**Conclusions:**

The meta-analysis suggested that the T allele of the rs1801133 polymorphism is a risk factor for CAD, which is possibly and partly mediated by abnormal lipid levels.

**Electronic supplementary material:**

The online version of this article (10.1186/s12944-018-0837-y) contains supplementary material, which is available to authorized users.

## Background

Coronary artery disease (CAD) is currently the leading cause of death in developed countries, and in some developing countries like China [1]. CAD is a multifactorial disease and a number of risk factors have been identified in the past few decades. Genetic polymorphism and dyslipidemia are the two most important risk factors for CAD [2, 3]. Genetic polymorphism is a term used to describe multiple forms of a single gene. Dyslipidemia is a state of abnormal amounts of lipids (e.g. triglycerides, cholesterol and/or phospholipids) in the blood, and is characterized by increased levels of triglycerides (TG), total cholesterol (TC) and low-density lipoprotein cholesterol (LDL-C), and/or decreased level of high-density lipoprotein cholesterol (HDL-C). Intensive efforts have been made in the scientific community to investigate the associations of the genetic polymorphisms in some specific genes with CAD risk and plasma lipid levels, but the results were inconsistent and inconclusive. It is difficult to identify the CAD- or dyslipidemia-related genetic polymorphisms successfully due to various reasons such as small sample sizes and ethnic differences.

5,10-methylenetetrahydrofolate reductase (MTHFR) is a rate-limiting enzyme in the one-carbon metabolism pathway and plays a key role in one-carbon metabolism by irreversibly catalyzing the conversion of 5,10-Methylenetetrahydrofolate (5,10-MTHF) to 5-Methyltetrahydrofolate (5-MTHF). 5-MTHF is a direct one-carbon donor (methyl group) for many substrates such as DNA [4], RNA [5] and proteins [6]. More importantly, 5-MTHF is the only one-carbon donor for the remethylation of homocysteine which is produced in methionine cycle. In methionine cycle, methionine first reacts with adenosine triphosphate to form S-adenosylmethionine (SAM) under the catalysis of methionine adenosyltransferase. The methyl group in SAM is activated, and SAM is called activated methionine. The activated methyl group of SAM can be transferred to target substrates such as DNA under the catalysis of methyltransferase, and SAM itself is converted into S-adenosine homocysteine after demethylation. Homocysteine is produced after the removal of adenosine from S-adenosine homocysteine under the catalysis of S-adenosylhomocysteine hydrolase. In the last step, homocysteine accepts the methyl group from 5-MTHF and methionine is formed again.

Homocysteine a potential risk factor for CAD and MTHFR has been reported to be associated with CAD risk and abnormal lipid levels [7–9]. Mikael et al. [8] reported that MTHFR(+/−) mice had significantly higher levels of plasma TG and more lipid deposition in aortic sinus compared with MTHFR (+/+) mice. In another study, Christensen et al. [9] found that high folic acid consumption led to pseudo-MTHFR deficiency in mice, and this deficiency resulted in altered lipid metabolism and liver injury.

The rs1801133 polymorphism (also known as the 677C > T polymorphism) is located in exon 4 of the *MTHFR* gene and formed by a transition from cytosine (C) to thymine (T). The 222nd genetic code of the *MTHFR* gene is changed accordingly from GCC to GTC, resulting in the replacement of alanine (Ala) by valine (Val) in the MTHFR polypeptide. A large number of studies have investigated the associations of the rs1801133 polymorphism with CAD and lipid levels. In some of these studies, the T allele of the rs1801133 polymorphism was reported to be associated with an increased risk of CAD [10–12] and elevated levels of TG [13, 14], TC [13–15] and LDL-C [13–16], and reduced levels of HDL-C [13, 17]. However, the results obtained from other studies did not support these findings [18–22]. Hence, a meta-analysis is required to clarify the relationships of the rs1801133 polymorphism with CAD and lipid levels.

Although two meta-analyses [23, 24] have addressed the issue of the association between the rs1801133 polymorphism and CAD in 2002 and 2005, respectively, their sample sizes were relatively small, and blood lipid variables were not considered in the analyses. In this study, a systematic review and updated meta-analysis was performed based on previous publications to investigate the associations of the rs1801133 polymorphism with CAD and lipid levels. The results of this meta-analysis can provide an opportunity to unveil the interrelationships among the rs1801133 polymorphism, dyslipidemia and susceptibility to CAD.

## Result

### Characteristics of the included studies

**Fig. 1:**
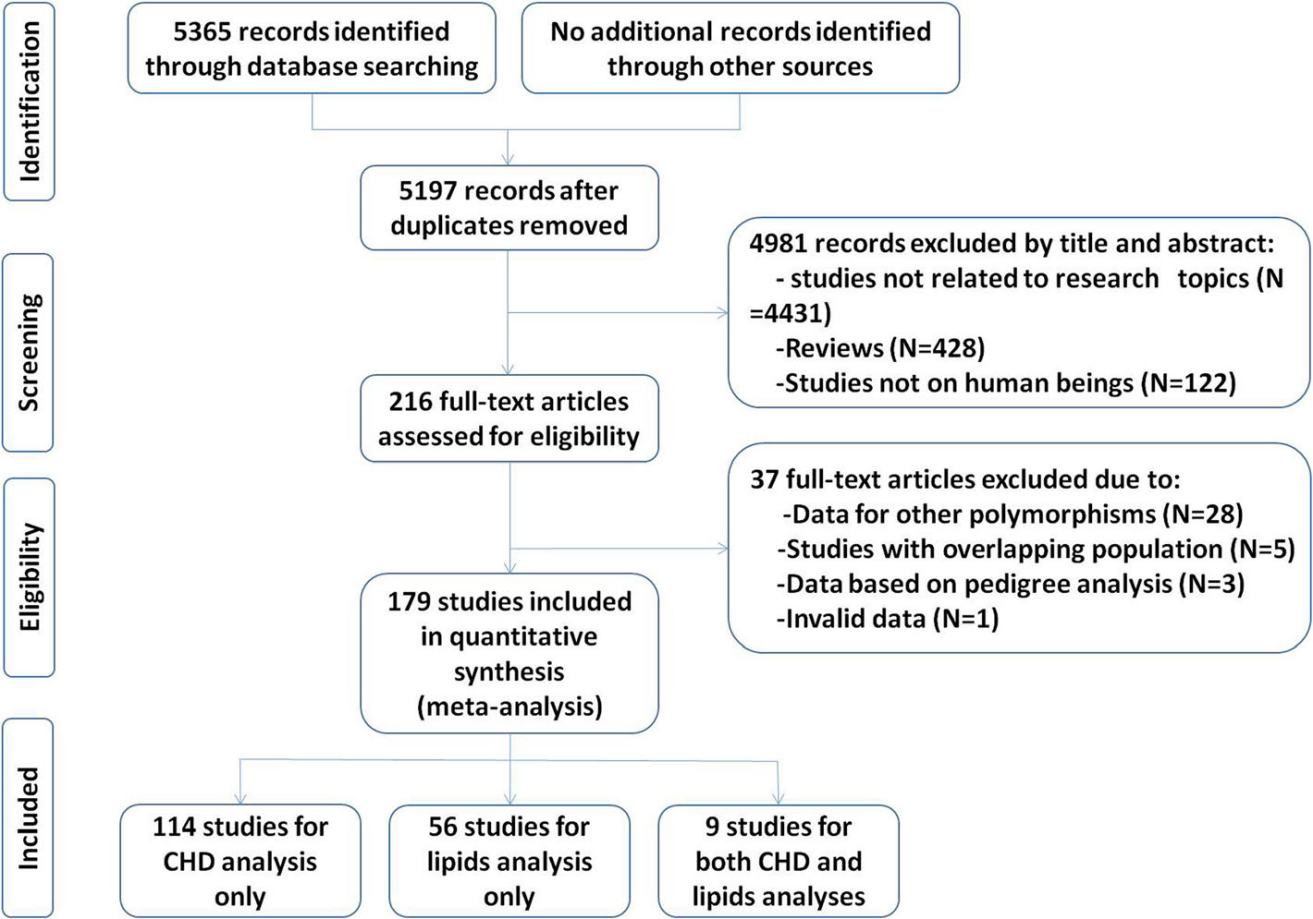
Flow diagram of the study selection process

Initial search of the databases yielded 5197 articles. Four thousand nine hundred and eighty-one studies were excluded according to the titles and abstracts. Then full-text articles were retrieved and assessed on the basis of inclusion criteria. Thirty-seven studies were ineligible for the following reasons: 28 studies presented data for other polymorphisms; 5 studies had subjects overlapping with other publications; 3 studies were based on pedigree analysis; 1 study presented invalid data. In the end, 179 studies were selected for this meta-analysis (Fig. 1). One hundred twenty-three studies (87,020 subjects) of them were included in the CAD association analysis, and 65 studies (85,554 subjects) were included in the lipid association analysis. The references for the studies included in the present meta-analysis are listed in Additional file 1.

The characteristics of the studies included in the CAD association analysis are summarized in Additional file 2: Table S1. Seventy-two studies, 35 studies, 5 studies and 11 studies involved in Caucasians, Asians, Africans and the subjects of other ethnic origins, respectively. The characteristics of the studies included in the lipid association analysis are summarized in Additional file 2: Table S2. The plasma lipid levels according to the genotypes of the rs1801133 polymorphism are presented in Additional file 2: Table S3. Twenty-four studies, 25 studies, 3 studies and 13 studies involved in Caucasians, Asians, Africans and the subjects of other ethnic origins, respectively. Eleven studies, 5 studies, 6 studies and 33 studies involved in CAD, diabetes, hypertension and healthy subjects, respectively. Fifty-three studies, 61 studies, 49 studies and 58 studies presented the data for TG, TC, LDL-C and HDL-C, respectively.

### Association of the r1801133 polymorphism with CAD

The variant T allele of the rs1801133 polymorphism was associated with increased risk of CAD under allelic model [odds ratio (OR) = 1.11, 95% confidence interval (CI) = 1.06–1.17, *P* < 0.01], additive model (OR = 1.25, 95% CI = 1.14–1.37, *P* < 0.01), dominant model (OR = 1.11, 95% CI = 1.04–1.17, *P* < 0.01) and recessive model (OR = 1.22, 95% CI = 1.12–1.32, *P* < 0.01) (Table 1). When the analyses were limited to the studies in Hardy-Weinberg equilibrium (HWE), the association between the rs1801133 polymorphism and CAD under allelic model (OR = 1.11, 95% CI = 1.06–1.16, *P* < 0.01), additive model (OR = 1.23, 95% CI = 1.12–1.35, *P* < 0.01), dominant model (OR = 1.11, 95% CI = 1.05–1.17, *P* < 0.01) and recessive model (OR = 1.20, 95% CI = 1.11–1.30, *P* < 0.01) was also significant (Table 1). Then the subgroup analyses stratified by the ethnicity of the subjects were performed, and the results showed that the rs1801133 polymorphism was strongly associated with CAD in Asians, but not in Africans or the subjects of other ethnic origins. In Caucasians, the association between the rs1801133 polymorphism and CAD was ambiguous, i.e. the rs1801133 polymorphism was marginally significantly associated with CAD under additive model (OR = 1.08, 95% CI = 1.00–1.17, *P* = 0.05) and recessive model (OR = 1.09, 95% CI = 1.01–1.17, *P* = 0.02), but not under allelic model and dominant model (Table 1).

**Table 1:** Meta-analysis between the *MTHFR* rs1801133 polymorphism and CAD risk

Model	Group or subgroup	OR	95% CI	*P*_heterogeneity_	*P*_OR_
Allelic model (T vs C)	Total	1.11	1.06–1.17	< 0.01	< 0.01
	Studies in HWE	1.11	1.06–1.16	< 0.01	< 0.01
	Male	1.04	0.90–1.20	< 0.01	0.59
	Female	0.98	0.88–1.08	0.40	0.63
	Caucasian	1.03	0.99–1.07	< 0.01	0.10
	Asian	1.24	1.11–1.39	< 0.01	< 0.01
	African	1.30	0.84–2.00	< 0.01	0.24
	Other ethnicity	1.30	0.96–1.76	< 0.01	0.09
Additive model (TT vs CC)	Total	1.25	1.14–1.37	< 0.01	< 0.01
	Studies in HWE	1.23	1.12–1.35	< 0.01	< 0.01
	Male	1.22	0.96–1.55	< 0.01	0.10
	Female	0.93	0.75–1.16	0.65	0.53
	Caucasian	1.08	1.00–1.17	0.03	0.05
	Asian	1.46	1.20–1.76	< 0.01	< 0.01
	African	1.96	0.84–4.60	< 0.01	0.12
	Other ethnicity	1.83	0.93–3.59	< 0.01	0.08
Dominant model (TT + CT vs CC)	Total	1.11	1.04–1.17	< 0.01	< 0.01
	Studies in HWE	1.11	1.05–1.17	< 0.01	< 0.01
	Male	1.00	0.80–1.25	< 0.01	0.98
	Female	0.98	0.83–1.16	0.20	0.84
	Caucasian	1.02	0.97–1.06	0.05	0.45
	Asian	1.27	1.08–1.50	< 0.01	< 0.01
	African	1.22	0.78–1.92	< 0.01	0.39
	Other ethnicity	1.30	0.95–1.77	< 0.01	0.10
Recessive model (TT vs CT + CC)	Total	1.22	1.12–1.32	< 0.01	< 0.01
	Studies in HWE	1.20	1.11–1.30	< 0.01	< 0.01
	Female	0.96	0.78–1.17	0.76	0.67
	Male	1.21	0.96–1.51	< 0.01	0.10
	Caucasian	1.09	1.01–1.17	0.06	0.02
	Asian	1.36	1.14–1.62	< 0.01	< 0.01
	African	1.90	0.84–4.30	< 0.01	0.13
	Other ethnicity	1.71	0.94–3.12	< 0.01	0.08

*MTHFR* 5,10-methylenetetrahydrofolate reductase gene, *CAD* coronary artery disease, *OR* odds ratio, *95% CI* 95% confidence interval, *HWE* Hardy-Weinberg equilibrium

### Associations of the r1801133 polymorphism with plasma lipid levels

**Fig. 2:**
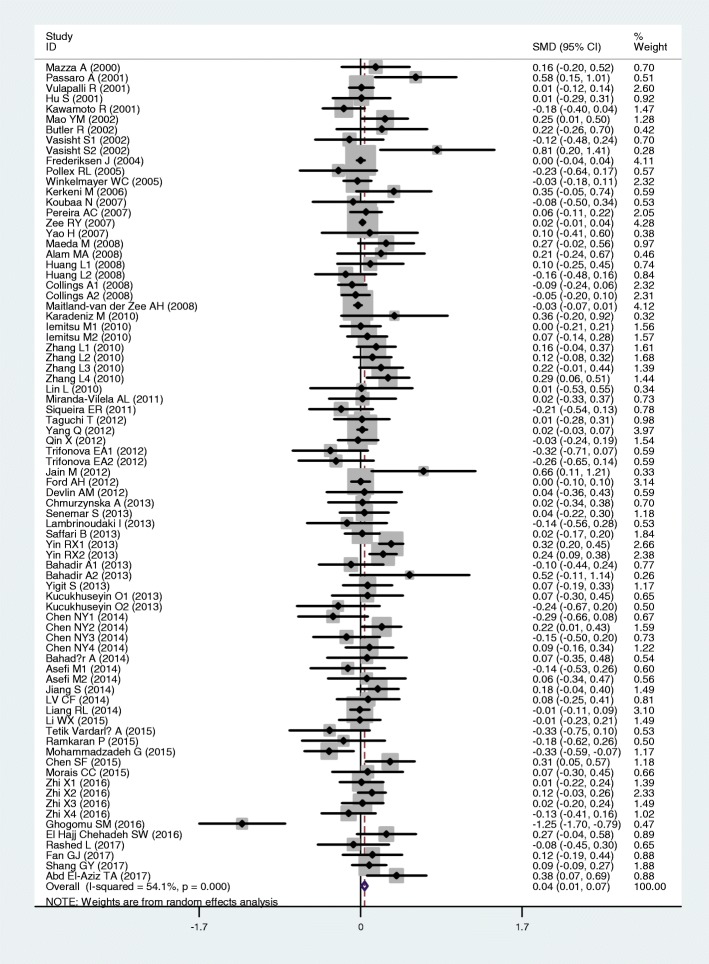
Forest plot of the meta-analysis between the *MTHFR* rs1801133 polymorphism and plasma total cholesterol (TC) levels

**Fig. 3:**
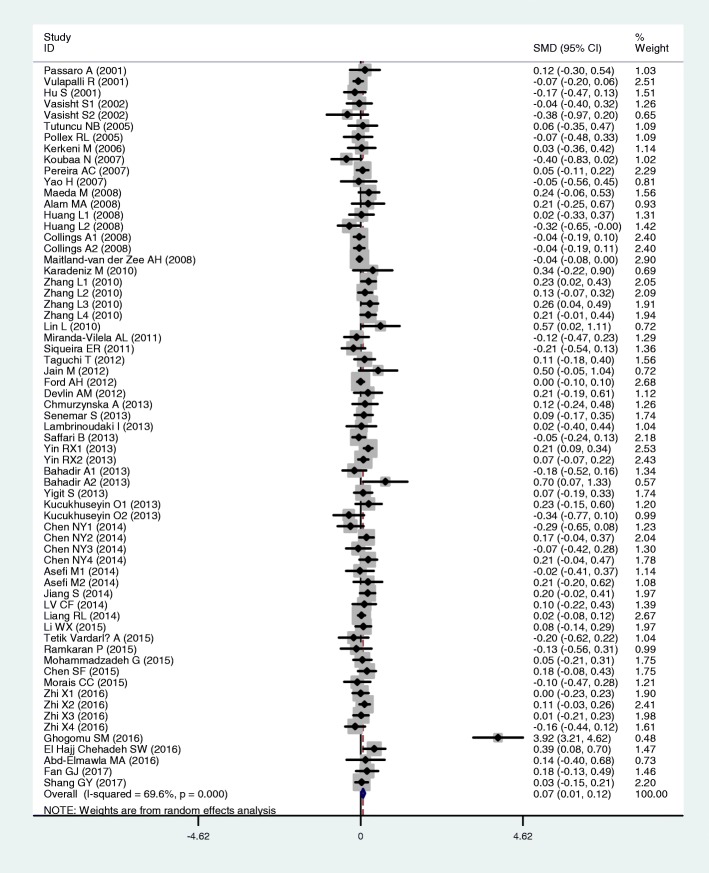
Forest plot of the meta-analysis between the *MTHFR* rs1801133 polymorphism and plasma low-density lipoprotein cholesterol (LDL-C) levels

**Fig. 4:**
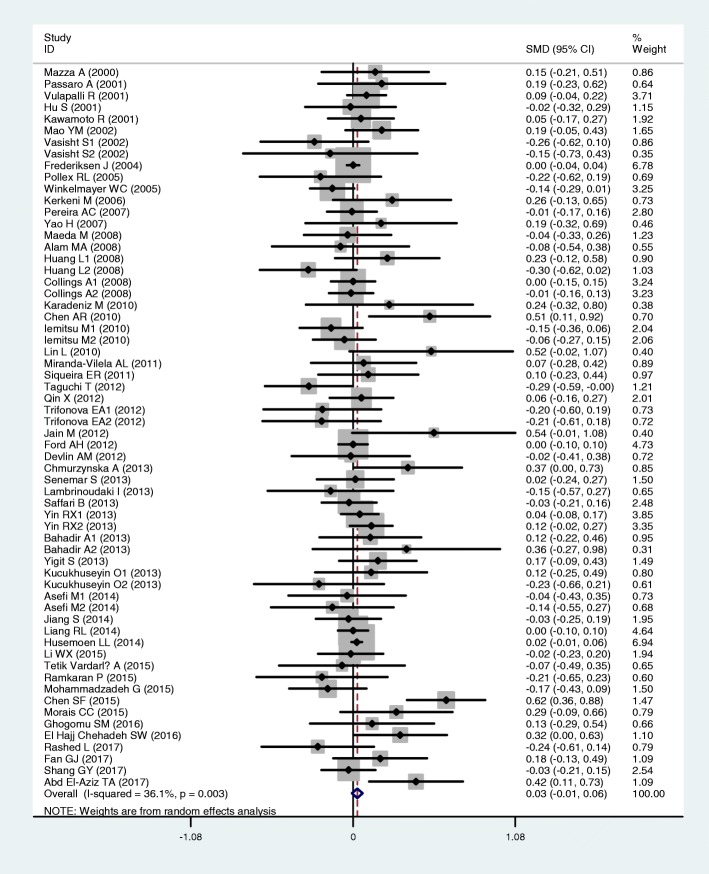
Forest plot of the meta-analysis between the *MTHFR* rs1801133 polymorphism and triglycerides (TG) levels

**Fig. 5:**
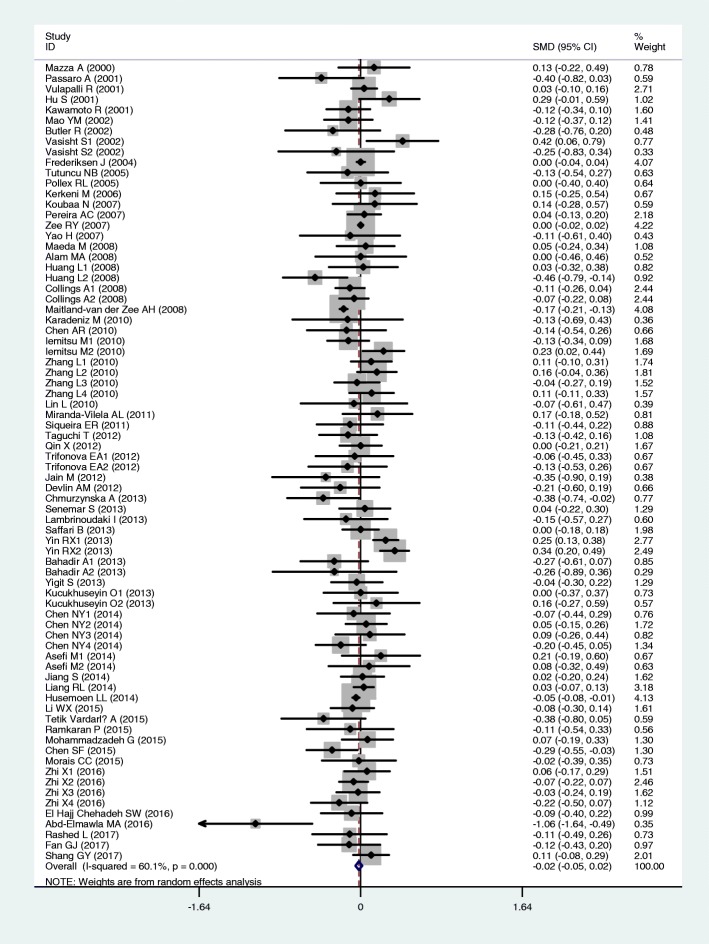
Forest plot of the meta-analysis between the *MTHFR* rs1801133 polymorphism and plasma high-density lipoprotein cholesterol (HDL-C) levels

The outcomes of the analyses on all comparisons showed that the T allele carriers had higher levels of TC [standardized mean difference (SMD) = 0.04, 95% CI = 0.01–0.07, *P* = 0.02] and LDL-C (SMD = 0.07, 95% CI = 0.01–0.12, *P* = 0.01) than the non-carriers (Table 2, Figs. 2 and 3), and that there were no associations detected between the rs1801133 polymorphism and plasma levels of TG (SMD = 0.03, 95% CI = − 0.01-0.06, *P* = 0.11) and HDL-C (SMD = − 0.02, 95% CI = − 0.05-0.02, *P* = 0.30) (Table 2, Figs. 4 and 5). When the analyses were limited to the studies in HWE, the T allele carriers had higher levels of LDL-C (SMD = 0.04, 95% CI = 0.01–0.08, *P* = 0.03) and lower levels of HDL-C (SMD = − 0.02, 95% CI = − 0.03--0.00, *P* = 0.04) than the non-carriers (Table 2).

**Table 2:** Meta-analysis between the *MTHFR* rs1801133 polymorphism and plasma lipid levels

Group or subgroup	Comparison (Subjects)	SMD (95% CI)	*P*_Heterogeneity_	*P*_SMD_
TG				
All	62 (39,760)	0.03 (−0.01–0.06)	< 0.01	0.11
Studies in HWE	46 (34,889)	0.01 (− 0.01–0.03)	0.11	0.44
Male	4 (2518)	0.00 (− 0.08–0.08)	0.71	0.96
Female	8 (1589)	0.13 (− 0.07–0.33)	0.01	0.21
Caucasian	25 (29,136)	0.00 (−0.04–0.04)	0.11	0.93
Asian	24 (8639)	0.05 (−0.02–0.13)	< 0.01	0.16
African	2 (191)	0.20 (−0.09–0.48)	0.65	0.18
Other ethnicity	11 (1794)	0.07 (−0.03–0.17)	0.79	0.15
CAD	10 (2907)	0.01 (−0.06–0.09)	0.54	0.74
T2DM	6 (812)	0.07 (−0.14–0.28)	0.09	0.51
Hypertension	4 (1522)	0.03 (−0.09–0.15)	0.70	0.63
Healthy or control	17 (26,612)	0.01 (−0.02–0.04)	0.33	0.50
TC				
All	79 (72,848)	0.04 (0.01–0.07)	< 0.01	0.02
Studies in HWE	57 (51,283)	0.01 (−0.00–0.03)	0.13	0.12
Male	11 (5067)	0.03 (−0.05–0.11)	0.18	0.52
Female	15 (28,790)	0.11 (0.02–0.19)	0.01	0.02
Caucasian	26 (41,744)	0.00 (−0.05–0.04)	0.02	0.95
Asian	36 (13,385)	0.10 (0.04–0.15)	0.01	< 0.01
African	3 (277)	−0.32 (−1.24–0.60)	< 0.01	0.49
Other ethnicity	14 (17,442)	−0.01 (− 0.04–0.02)	0.48	0.55
CAD	9 (2676)	0.02 (−0.06–0.10)	0.45	0.66
T2DM	5 (705)	0.03 (−0.20–0.27)	0.09	0.77
Hypertension	6 (11,288)	−0.02 (− 0.06–0.02)	0.47	0.34
Healthy or control	31 (49,859)	0.07 (0.02–0.12)	< 0.01	< 0.01
LDL-C				
All	65 (29,424)	0.07 (0.01–0.12)	< 0.01	0.01
Studies in HWE	45 (13,946)	0.04 (0.01–0.08)	0.14	0.03
Male	10 (4999)	0.04 (−0.04–0.12)	0.13	0.36
Female	13 (3662)	0.09 (0.01–0.16)	0.43	0.02
Caucasian	18 (6226)	0.00 (−0.05–0.05)	0.71	0.95
Asian	31 (11,417)	0.10 (0.05–0.15)	0.10	< 0.01
African	3 (277)	1.16 (−0.94–3.25)	< 0.01	0.28
Other ethnicity	13 (11,504)	−0.02 (− 0.10–0.06)	0.23	0.67
CAD	9 (2807)	0.00 (−0.08–0.07)	0.77	0.92
T2DM	6 (799)	0.01 (−0.25–0.26)	0.02	0.96
Hypertension	5 (10,843)	0.06 (−0.05–0.18)	0.12	0.30
Healthy or control	25 (8989)	0.07 (0.01–0.13)	0.02	0.02
HDL-C				
All	76 (76,811)	−0.02 (− 0.05–0.02)	< 0.01	0.30
Studies in HWE	57 (61,438)	−0.02 (− 0.03--0.00)	0.72	0.04
Male	10 (3467)	−0.04 (− 0.11–0.03)	0.79	0.29
Female	14 (28,630)	−0.05 (− 0.12–0.02)	0.10	0.19
Caucasian	25 (51,768)	−0.03 (− 0.07–0.01)	0.02	0.16
Asian	25 (9553)	0.00 (−0.06–0.06)	< 0.01	0.92
African	2 (186)	0.15 (− 0.14–0.43)	0.99	0.32
Other ethnicity	3 (282)	−0.10 (− 0.18--0.02)	0.26	0.01
CAD	8 (2570)	0.05 (− 0.04–0.13)	0.65	0.27
T2DM	6 (799)	0.01 (−0.14–0.16)	0.88	0.89
Hypertension	4 (1522)	−0.03 (− 0.15–0.08)	0.85	0.56
Healthy or control	32 (56,554)	0.00 (− 0.05–0.04)	< 0.01	0.97

*MTHFR* 5,10-methylenetetrahydrofolate reductase gene, *SMD* standardized mean difference, *95% CI* 95% confidence interval, *HWE* Hardy-Weinberg equilibrium, *TG* triglyceride, *TC* total cholesterol, *LDL-C* low-density lipoprotein cholesterol, *HDL-C* high-density lipoprotein cholesterol, *CAD* coronary artery disease, *T2DM* type 2 diabetes mellitus

Then the subgroup analyses stratified by the characteristics of the subjects were performed. The significant associations of the rs1801133 polymorphism with higher levels of TC (SMD = 0.11, 95% CI = 0.02–0.19, *P* = 0.02) and LDL-C (SMD = 0.09, 95% CI = 0.01–0.16, *P* = 0.02) were detected in females, but not in males. The significant associations of the rs1801133 polymorphism with higher levels of TC (SMD = 0.10, 95% CI = 0.04–0.15, *P* < 0.01) and LDL-C (SMD = 0.10, 95% CI = 0.05–0.15, *P* < 0.01) were detected in Asians, but not in the subjects of other ethnic origins. However, the rs1801133 polymorphism was significantly associated with lower level of HDL-C (SMD = − 0.10, 95% CI = − 0.18--0.02, *P* = 0.01) only in the subjects of other ethnic origins. When health status was taken into account, the significant associations of the rs1801133 polymorphism with higher levels of TC (SMD = 0.07, 95% CI = 0.02–0.12, *P* < 0.01) and LDL-C (SMD = 0.07, 95% CI = 0.01–0.13, *P* = 0.02) were detected in healthy or control subjects, but not in the patients with CAD, type 2 diabetes mellitus (T2DM) and hypertension (Table 2).

### Heterogeneity analysis

In the CAD association analysis, there was significant heterogeneity in allelic model (*I*^*2*^ = 68.2%, *P*_*heterogeneity*_ < 0.01), additive model (*I*^*2*^ = 55.6%, *P*_*heterogeneity*_ < 0.01), dominant model (*I*^2^ = 64.0%, *P*_*heterogeneity*_ < 0.01) and recessive model (*I*^*2*^ = 51.5%, *P*_*heterogeneity*_ < 0.01). Twenty-seven studies, 22 studies, 19 studies and 17 studies were identified as the main contributors to the heterogeneity for allelic model, additive model, dominant model and recessive model, respectively, by using Galbraith plots. The heterogeneity was effectively removed or decreased after exclusion of these outlier studies, but OR values and 95% CIs did not change substantially (allelic model: OR = 1.04, 95% CI = 1.01–1.07, *P*_*heterogeneity*_ = 0.12, *P*_*OR*_ = 0.01; additive model: OR = 1.12, 95% CI = 1.05–1.19, *P*_*heterogeneity*_ = 0.09, *P*_*OR*_ < 0.01; dominant model: OR = 1.04, 95% CI = 1.00–1.07, *P*_*heterogeneity*_ = 0.14, *P*_*OR*_ = 0.05; recessive model: OR = 1.12, 95% CI = 1.06–1.18, *P*_*heterogeneity*_ = 0.13, *P*_*OR*_ < 0.01) (Table 3).

**Table 3:** Meta-analysis between the *MTHFR* rs1801133 polymorphism and CAD risk after excluding the studies with heterogeneity

Comparisons	Ethnicity	OR	95% CI	*P*_heterogeneity_	*P*_OR_
Allelic model (T vs C)	Total	1.04	1.01–1.07	0.12	0.01
	Male	1.04	0.94–1.14	0.13	0.48
	Female	1.04	0.93–1.16	0.80	0.53
	Caucasian	1.03	1.00–1.06	0.32	0.07
	Asian	1.11	1.04–1.18	0.32	< 0.01
	African	0.94	0.78–1.13	0.11	0.50
	Other ethnicity	0.97	0.83–1.14	0.13	0.74
Additive model (TT vs CC)	Total	1.12	1.05–1.19	0.09	< 0.01
	Male	1.07	0.91–1.27	0.23	0.42
	Female	1.02	0.79–1.31	0.82	0.87
	Caucasian	1.08	1.01–1.16	0.57	0.03
	Asian	1.28	1.12–1.46	0.05	< 0.01
	African	0.79	0.50–1.26	0.26	0.32
	Other ethnicity	1.23	0.91–1.66	0.10	0.17
Dominant model (TT + CT vs CC)	Total	1.04	1.00–1.07	0.14	0.05
	Male	1.04	0.94–1.15	0.16	0.44
	Female	1.07	0.91–1.25	0.51	0.42
	Caucasian	1.01	0.97–1.05	0.61	0.56
	Asian	1.15	1.05–1.25	0.14	< 0.01
	African	1.01	0.81–1.26	0.07	0.94
	Other ethnicity	1.04	0.86–1.26	0.10	0.67
Recessive model (TT vs CT + CC)	Total	1.12	1.06–1.18	0.13	< 0.01
	Male	1.15	0.99–1.33	0.05	0.06
	Female	0.95	0.77–1.17	0.76	0.62
	Caucasian	1.07	1.01–1.14	0.68	0.03
	Asian	1.28	1.14–1.43	0.06	< 0.01
	African	0.78	0.52–1.18	0.29	0.24
	Other ethnicity	1.22	0.93–1.60	0.18	0.16

*MTHFR* 5,10-methylenetetrahydrofolate reductase gene, *CAD* coronary artery disease, *OR* odds ratio, *95% CI* 95% confidence interval

In the lipid association analysis, there was significant heterogeneity in the total comparisons for TG (*I*^*2*^ = 36.1%, *P*_*heterogeneity*_ < 0.01), TC (*I*^*2*^ = 54.1%, *P*_*heterogeneity*_ < 0.01), LDL-C (*I*^*2*^ = 69.6%, *P*_*heterogeneity*_ < 0.01) and HDL-C (*I*^*2*^ = 60.1%, *P*_*heterogeneity*_ < 0.01). Three comparisons, 9 comparisons, 3 comparisons and 6 comparisons were identified as the main contributors to the heterogeneity for TG, TC, LDL-C and HDL-C, respectively, by using Galbraith plots. SMD values and 95% CIs of TG and LDL-C did not change substantially after excluding the outlier comparisons. However, SMD values and 95% CIs of TC (SMD = 0.01, 95% CI = − 0.00-0.03, *P*_*heterogeneity*_ = 0.38, *P*_*SMD*_ = 0.15) and HDL-C (SMD = − 0.02, 95% CI = − 0.03--0.00, *P*_*heterogeneity*_ = 0.48, *P*_*SMD*_ = 0.05) changed significantly after excluding these outlier comparisons (Table 4).

**Table 4:** Meta-analysis between the *MTHFR* rs1801133 polymorphism and plasma lipid levels after excluding the comparisons with heterogeneity

Group or subgroup	Comparison (Subjects)	SMD (95% CI)	*P*_Heterogeneity_	*P*_SMD_
TG				
All	59 (39,196)	0.01 (−0.01–0.03)	0.32	0.22
Male	4 (2518)	0.00 (− 0.08–0.08)	0.71	0.96
Female	7 (1429)	0.02 (− 0.09–0.13)	0.06	0.74
Caucasian	24 (28,976)	0.01 (− 0.02–0.03)	0.31	0.54
Asian	22 (8235)	0.02 (−0.03–0.06)	0.17	0.50
African	2 (191)	0.20 (−0.09–0.48)	0.65	0.18
Other ethnic	11 (1794)	0.07 (−0.03–0.17)	0.79	0.15
CAD	10 (2907)	0.01 (−0.06–0.09)	0.54	0.74
T2DM	6 (812)	0.07 (−0.08–0.22)	0.09	0.35
Hypertension	4 (1522)	0.03 (−0.09–0.15)	0.70	0.63
Healthy or control	17 (26,612)	0.01 (−0.01–0.04)	0.33	0.28
TC				
All	70 (70,024)	0.01 (−0.00–0.03)	0.38	0.15
Male	11 (5067)	0.02 (−0.04–0.08)	0.18	0.44
Female	11 (28, 074)	0.02 (−0.00–0.04)	0.54	0.09
Caucasian	22 (41,176)	0.01 (−0.01–0.03)	0.81	0.42
Asian	32 (11,220)	0.06 (0.02–0.10)	0.34	< 0.01
African	2 (186)	0.15 (−0.14–0.44)	0.15	0.31
Other ethnic	14 (17,442)	−0.01 (− 0.04–0.02)	0.48	0.55
CAD	10 (2907)	0.02 (−0.06–0.10)	0.45	0.66
T2DM	5 (705)	0.07 (−0.09–0.23)	0.09	0.38
Hypertension	6 (11,288)	−0.02 (− 0.06–0.02)	0.47	0.34
Healthy or control	26 (47,611)	0.02 (−0.00–0.04)	0.36	0.08
LDL-C				
All	62 (18,739)	0.04 (0.01–0.07)	0.10	0.01
Male	10 (4999)	0.04 (−0.02–0.10)	0.13	0.24
Female	13 (3662)	0.09 (0.01–0.16)	0.43	0.02
Caucasian	18 (6226)	0.00 (−0.05–0.05)	0.71	0.95
Asian	30 (10,439)	0.08 (0.04–0.12)	0.16	< 0.01
African	2 (186)	−0.17 (−0.46–0.12)	0.14	0.25
Other ethnic	12 (1888)	0.00 (−0.10–0.09)	0.19	0.98
CAD	9 (2807)	0.00 (−0.08–0.07)	0.77	0.92
T2DM	6 (799)	0.06 (−0.09–0.21)	0.02	0.43
Hypertension	4 (1227)	0.14 (0.01–0.27)	0.87	0.03
Healthy or control	24 (8011)	0.06 (0.01–0.11)	0.06	0.01
HDL-C				
All	70 (65,091)	−0.02 (−0.03--0.00)	0.48	0.05
Male	10 (3467)	−0.04 (− 0.11–0.03)	0.79	0.29
Female	14 (28,630)	−0.01 (− 0.03–0.02)	0.10	0.61
Caucasian	23 (51,561)	−0.02 (− 0.03–0.00)	0.49	0.08
Asian	33 (11,456)	−0.02 (− 0.06–0.02)	0.22	0.42
African	2 (186)	0.15 (−0.14–0.43)	0.99	0.32
Other ethnic	12 (1888)	−0.04 (− 0.13–0.05)	0.70	0.41
CAD	9 (2766)	0.03 (−0.06–0.11)	0.99	0.54
T2DM	6 (799)	0.01 (−0.14–0.16)	0.88	0.89
Hypertension	4 (1522)	−0.03 (− 0.15–0.08)	0.85	0.56
Healthy or control	30 (54,825)	−0.02 (− 0.03–0.00)	0.05	0.06

*MTHFR* 5,10-methylenetetrahydrofolate reductase gene, *SMD* standardized mean difference, *95% CI* 95% confidence interval, *TG* triglyceride, *TC* total cholesterol, *LDL-C* low-density lipoprotein cholesterol, *HDL-C* high-density lipoprotein cholesterol, *CAD* coronary artery disease, *T2DM* type 2 diabetes mellitus

### Publication bias test

Begg’s test and Egger’s test were used to evaluate the publication bias of the included studies, and the results showed that there might be a publication bias in the analysis between the rs1801133 polymorphism and CAD (*P* < 0.05 for all genetic models). To clarify this problem, a trim-and-fill method was used to adjust the results, and no trimming was performed and the results were unchanged. It indicates that there is no publication bias in the literature. The significant *P* values of Begg’s test and Egger’s test were originated from other factors, e.g. mixed ethnicity in some studies. No publication bias was detected in the lipid association analysis.

## Discussion

In the present meta-analysis, the variant T allele of the rs1801133 polymorphism was associated with increased risk of CAD, and elevated levels of TC and LDL-C in the total population. It indicates that the T allele of the rs1801133 polymorphism is a risk factor for CAD, which is at least partly mediated by abnormal lipid levels.

A large number of studies have investigated the association between the rs1801133 polymorphism and CAD risk, as well as the underlying mechanisms, but most of them just focused on homosysteine. It was widely reported that the rs1801133 polymorphism influences the plasma levels of homosysteine in various populations such as Americans [25], Africans [17, 26, 27], Asians [18, 28, 29], Turkish [30, 31] and Brazilians [32]. In methionine cycle, homocysteine is formed after the removal of adenosine from S-adenosine homocysteine. Under normal condition, homocysteine is remethylated to methionine by accepting a methyl group from 5-MTHF. 5-MTHF is formed from the reduction of 5,10-MTHF under the catalysis of MTHFR. In the T allele carriers of the rs1801133 polymorphism, the function of MTHFR may be affected since the normal alanine residue is replaced by a valine residue in the polypeptide, which in turn affects the production of 5-MTHF and the remethylation of homcysteine, resulting in elevated plasma homosysteine levels. Studies have shown that homocysteine is a risk factor for CAD, and oxidative stress [33], vascular inflammation [34] and endothelial injury [35] are involved in the underlying mechanisms in which homocysteine causes CAD. All these events are likely to trigger the development of atherosclerosis and arterial thrombosis.

However, the role of homocysteine in the pathogenesis of CAD is controversial. Several studies [36–38] demonstrated no association between homocysteine and CAD. It indicates that some other risk factors are involved in the association between the rs1801133 polymorphism and CAD. In this meta-analysis, the results showed that the variant T allele carriers of the rs1801133 polymorphism have higher levels of TC and LDL-C than the non-carriers, which indicates that the abnormal lipid levels caused by the T allele of the rs1801133 polymorphism might be one of the important reasons in the development of CAD since dyslipidemia is closely associated with the progression of coronary atherosclerosis, and it accounts for around 50% of the population-attributable risk for CAD [39]. According to the 2013 ACC/AHA blood cholesterol guidelines [40] and the Adult Treatment Panel III (ATP III) Guidelines [41] of the United States, LDL-C was considered as a major cause of CAD and used as the primary target for therapy, and other lipid parameters were used as the secondary or supplementary targets.

The mechanisms in which the rs1801133 polymorphism is associated with plasma lipid levels have not been clarified yet. Several possible reasons can be proposed to explain the association between the rs1801133 polymorphism and plasma lipid levels. Firstly, the rs1801133 polymorphism may indirectly affect plasma lipid levels through the mediation of homocysteine [42–44]. Baszczuk et al. [42] reported that a daily administration of 15 mg of folic acid led to a considerable decrease in homocysteine levels, and a substantive increase in HDL-C levels in the patients with primary hypertension. In yeast cells, homocysteine supplementation increased cellular fatty acid and TG contents, induced a shift in fatty acid composition, and decreased the condensing enzymes involved in very long-chain fatty acid synthesis [43]. Secondly, the rs1801133 polymorphism may modulate the lipid metabolism by affecting the methylation state of DNA or proteins. 5-MTHF is not only the methyl donor for homocysteine, but for many other target molecules, including DNA and proteins [45]. Conceivably, the functions of the genes or proteins involved in lipid metabolism will be affected if their methylation state changes.

In most of the studies included in the lipid association analysis, a dominant model was used, i.e. TT + CT vs. CC. Therefore, a dominant model was also adopted in this meta-analysis. In subgroup analyses, we found that the differences in TC and LDL-C levels between the genotypes were mainly from Asian populations, whose SMD values were larger than those calculated in Caucasians, Africans and the subjects of other ethnicities (Table 2). The associations of the rs1801133 polymorphism with TC and LDL-C in Asians were consistently larger, which shows that there is a stronger association between the rs1801133 polymorphism and CAD in Asians as compared with other ethnicities (Table 1). Studies will be needed to elucidate the mechanisms that the rs1801133 polymorphism has different effects on blood lipid levels and CAD risk in different ethnicities.

In the lipid association analysis, subgroup analyses by gender and health status was performed since there might be important factors affecting the associations between the rs1801133 polymorphism and lipid levels. For example, the present meta-analysis indicates that gender might modulate the associations of the rs1801133 polymorphism with TC and LDL-C levels since there were significant associations existing only in females but not in males (Table 2). Health status might also modulate the associations of the rs1801133 polymorphism with TC and LDL-C levels. The significant associations of the rs1801133 polymorphism with TC and LDL-C levels only existed in healthy subjects, but not in the patients with CAD, T2DM and hypertension. The reason might be that the patients with CAD, T2DM and hypertension had serious metabolic disorders, which masked the effects of the rs1801133 polymorphism on TC and LDL-C levels. As compared with the diseases such as CAD, T2DM and hypertension, genetic polymorphisms usually have less impact on plasma lipid levels. In line with the results from the present study, several studies also reported that the rs1801133 polymorphism is associated TC and LDL-C levels in healthy subjects [13, 15, 46], but not in the patients with CAD [11, 20], T2DM [47, 48] and hypertension [49, 50]. Of the 179 studies included, 141 studies used polymerase chain reaction-restriction fragment length polymorphism (PCR-RFLP) method; 27 studies used real-time PCR method; 7 studies used DNA sequencing method; 1 study used gene chip method; and 3 studies did not report the genotyping method(s). Subgroup analyses stratified by the genotyping methods were conducted, and the results showed that there were differences in OR or SMD values among the studies with different genotyping methods (data not shown). In most cases, the results from the studies by PCR-RFLP method were in line with the results from all studies. The reason might be that most of the studies used PCR-RFLP method. The results from the studies by real-time PCR method or DNA sequencing method could have been affected by the small number of studies and small sample sizes.

Significant heterogeneity was detected in the total and subgroup analyses between the rs1801133 polymorphism and CAD, and the outlier studies were identified by using the Galbraith plots. No significant changes in OR values and 95% CIs were found after excluding the outlier studies (Table 3), which indicates that the association between the rs1801133 polymorphism and CAD is very strong. Significant heterogeneity was also detected in the total and some of the subgroup analyses between the rs1801133 polymorphism and plasma lipid levels. The outlier studies were identified and excluded, but SMD values and 95% CIs of LDL-C were not significantly changed in the total population or in Asians, which indicates that there is a strong association between the rs1801133 polymorphism and LDL-C levels, especially in Asians.

The associations of the rs1801133 polymorphism with CAD and plasma lipid levels are not likely to be type I errors (false-positive results). Firstly, the results from this meta-analysis are based on four different models for CAD association analysis, and on random effects model for lipid association analysis if the heterogeneity among the studies is significant (*I*^2^ > 50%). As compared with fixed effects model, the random effects model is a more conservative method and less likely to produce false-positive results. Secondly, 87,020 subjects and 85,554 subjects were included in the analysis for the CAD association analysis and the lipid association analysis, respectively. Among the subjects, 44.0% (CAD association analysis) and 52.3% (lipid association analysis) of them are the carriers of the variant T allele. Since the incidence of the T allele carriers is very high, type I errors could have been prevented in both the CAD and lipid association analyses. Thirdly, this meta-analysis only included the studies published in English and Chinese as it was very difficult to get the full papers published in various languages.

## Conclusions

The current meta-analysis demonstrates that the rs1801133 polymorphism is associated with increased risk of CAD and elevated levels of TC and LDL-C. Further studies will be needed to elucidate the underlying mechanisms in which the rs1801133 polymorphism affects plasma lipid levels.

## Methods

### Literature search

The articles published before September 2017 on the associations of the rs1801133 polymorphism with CAD and/or plasma lipid levels were identified. The languages of the articles were limited to English and Chinese. A comprehensive search was conducted and nine electronic databases were searched to identify all relevant articles. The databases are as follows: PubMed, Embase, Baidu Scholar, Google Scholar, Web of Science, Cochrane Library, Wanfang, CBM and CNKI. The following keywords were used: (“5,10-Methylenetetrahydrofolate reductase” or “Methylenetetrahydrofolate reductase” or “MTHFR”), (“polymorphism” or “mutation” or “variant” or “C677T” or “rs1801133” or “Ala222Val”), (“coronary artery disease” or “coronary heart disease” or “heart disease” or “coronary disease” or “cardiovascular disease” or “angina pectoris” or “acute coronary syndrome” or “myocardial infarction” or “CAD” or “CHD” or “HD” or “CD” or “AP” or “ACS” or “MI”), (“plasma lipid” or “blood lipid” or “serum lipid”).

### Inclusion and exclusion criteria

The inclusion criteria for the association analysis between the rs1801133 polymorphism and CAD are as follows: 1) studies using a population-based case-control design; 2) CAD cases were angiographically defined; 3) number or frequency of cases according to the r1801133 genotypes was available. The inclusion criteria for the association analysis between the rs1801133 polymorphism and lipid levels are as follows: 1) studies in which mean lipids and standard deviations (SD) or standard errors (SE) by the rs1801133 genotypes were available; 2) studies which reported at least one of the four lipid variables, i.e. TG, TC, LDL-C and HDL-C; 3) baseline data were used for interventional studies. All references cited by the included articles were reviewed to check the published work which was not indexed by PubMed, Embase, Baidu Scholar, Google Scholar, Web of Science, Cochrane Library, Wanfang, CBM and CNKI. Reports with incomplete data, studies based on pedigree data, case reports, review articles, abstracts and animal studies were excluded from the meta-analysis.

### Data extraction

Data were extracted from each study by using a structured data collection form and by two investigators independently according to the pre-specified selection criteria. Decisions were compared and disagreements about study selection were resolved by consensus or by involving a third investigator. For the overlapping articles, only the publications that presented the most detailed information were included. In this meta-analysis, the data extracted from each of the included studies are as follows: first author, year of publication, age, ethnicity, gender, health status, type of study, genotyping method, lipid assay method, sample size, and mean with SD or SE according to the r1801133 genotypes. If data in a study were unconvincing, we attempted to contact the corresponding or first author by e-mail and telephone.

### Data analysis

Statistical analysis was performed by using STATA version 12.0 (Stata Corporation LP, College Station, TX, USA). All the tests were two-sided and a *P*-value of less than 0.05 for any test or model was considered to be statistically significant. OR with 95% CI was used to evaluate the strength of the association between the rs1801133 polymorphism and CAD. The pooled OR was performed for allelic model (T vs C), additive model (TT vs CC), dominant model (TT + CT vs CC) and recessive model (TT vs CT + CC). SMD with 95% CI was used to assess the strength of the associations between the rs1801133 polymorphism and plasma lipid levels. A fixed-effect model (Mantel-Haenszel method) was used to evaluate the results if heterogeneity among the included studies was not significant (*I*^2^ < 50%). Otherwise, the random-effect model (DerSimonian-Laird method) was used [51]. Heterogeneity was investigated by Cochran’s χ^2^-based Q-statistic, and Galbraith plots were used to detect the potential sources of heterogeneity. OR and SMD values were recalculated after excluding the outlier studies. Subgroup analyses were performed according to ethnicity for CAD association analysis, and according to ethnicity, gender and health status for lipid association analysis. Ethnic subgroups were defined as Caucasian, Asian, African, and the subjects of other ethnic origin. Health status was defined as CAD, T2DM and hypertension. HWE was assessed by Fisher’s exact test. OR and SMD values were recalculated after excluding the studies which were not in HWE. Publication bias was tested by Begg’s funnel plots and Egger’s test [52].

## Additional files

Additional file 1: The reference list for the studies included in the present meta-analysis. (DOC 106 kb)

Additional file 2: **Table S1.** Characteristics of the individual studies included in the meta-analysis between the *MTHFR* rs1801133 polymorphism and CAD; **Table S2.** Characteristics of the individual studies included in the meta-analysis between the *MTHFR* rs1801133 polymorphism and plasma lipid levels; **Table S3.** Plasma lipid levels according to the genotypes of the *MTHFR* rs1801133 polymorphism. (DOC 544 kb)

## Abbreviations

95% CI: 95% confidence interval
CAD: coronary artery disease
HDL-C: high-density lipoprotein cholesterol
HWE: Hardy-Weinberg equilibrium
LDL-C: low-density lipoprotein cholesterol
SMD: standardized mean difference
T2DM: type 2 diabetes mellitus
TC: total cholesterol
TG: triglycerides
