# Supplementary material for: Associations of the MTHFR rs1801133 polymorphism with coronary artery disease and lipid levels: a systematic review and updated meta-analysis
Source: Lipids Health Dis. 2018 Aug 17;17:191. doi: 10.1186/s12944-018-0837-y (PMC6097444; doi:10.1186/s12944-018-0837-y)
Supplement: Supplementary file 1 — The reference list for the studies included in the present meta-analysis. (DOC 106 kb) [file 12944_2018_837_MOESM1_ESM.doc]

**The reference list for the studies included in the present meta-analysis**

**R1.** Wilcken DE, Wang XL, Sim AS, McCredie RM. Distribution in healthy and coronary populations of the methylenetetrahydrofolate reductase (MTHFR) C677T mutation. Arterioscler Thromb Vasc Biol. 1996; 16(7): 878-82.

**R2.** Ma J, Stampfer MJ, Hennekens CH, Frosst P, Selhub J, Horsford J, Malinow MR, Willett WC, Rozen R. Methylenetetrahydrofolate reductase polymorphism, plasma folate, homocysteine, and risk of myocardial infarction in US physicians. Circulation. 1996; 94(10): 2410-6.

**R3.** Adams M, Smith PD, Martin D, Thompson JR, Lodwick D, Samani NJ. Genetic analysis of thermolabile methylenetetrahydrofolate reductase as a risk factor formyocardial infarction. QJM. 1996; 89(6): 437-44.

**R4**. Schmitz C, Lindpaintner K, Verhoef P, Gaziano JM, Buring J. Genetic polymorphism of methylenetetrahydrofolate reductase and myocardial infarction. A case-control study. Circulation. 1996; 94(8): 1812-4.

**R5.** Izumi M, Iwai N, Ohmichi N, Nakamura Y, Shimoike H, Kinoshita M. Molecular variant of 5,10-methylenetetrahydrofolate reductase is a risk factor of ischemic heart disease in the Japanese population. Atherosclerosis. 1996; 121(2): 293-4.

**R6.** Gallagher PM, Meleady R, Shields DC, Tan KS, McMaster D, Rozen R, Evans A, Graham IM, Whitehead AS. Homocysteine and risk of premature coronary heart disease. Evidence for a common gene mutation. Circulation. 1996; 94(9): 2154-8.

**R7.** Brulhart MC, Dussoix P, Ruiz J, Passa P, Froguel P, James RW. The (Ala-Val) mutation of methylenetetrahydrofolate reductase as a genetic risk factor forvascular disease in non-insulin-dependent diabetic patients. Am J Hum Genet. 1997; 60(1): 228-9.

**R8.** Schwartz SM, Siscovick DS, Malinow MR, Rosendaal FR, Beverly RK, Hess DL, Psaty BM, Longstreth WT Jr, Koepsell TD, Raghunathan TE, Reitsma PH. Myocardial infarction in young women in relation to plasma total homocysteine, folate, and a common variant in the methylenetetrahydrofolate reductase gene. Circulation. 1997; 96(2): 412-7.

**R9.** Brugada R, Marian AJ. A common mutation in methylenetetrahydrofolate reductase gene is not a major risk of coronary artery disease or myocardial infarction. Atherosclerosis. 1997; 128(1): 107-12.

**R10.** van Bockxmeer FM, Mamotte CD, Vasikaran SD, Taylor RR. Methylenetetrahydrofolate reductase gene and coronary artery disease. Circulation. 1997; 95(1): 21-3.

**R11.** Anderson JL, King GJ, Thomson MJ, Todd M, Bair TL, Muhlestein JB, Carlquist JF. A mutation in the methylenetetrahydrofolate reductase gene is not associated with increased risk for coronary artery disease or myocardial infarction. J Am Coll Cardiol. 1997; 30(5): 1206-11.

**R12.** Kluijtmans LA, Kastelein JJ, Lindemans J, Boers GH, Heil SG, Bruschke AV, Jukema JW, van den Heuvel LP, Trijbels FJ, Boerma GJ, Verheugt FW,Willems F, Blom HJ. Thermolabile methylenetetrahydrofolate reductase in coronary artery disease. Circulation. 1997; 96(8): 2573-7.

**R13.** Morita H, Taguchi J, Kurihara H, Kitaoka M, Kaneda H, Kurihara Y, Maemura K, Shindo T, Minamino T, Ohno M, Yamaoki K, Ogasawara K, Aizawa T,Suzuki S, Yazaki Y. Gene Polymorphism of 5, 10-methylenetetrahydrofolate reductase as a coronary risk factor. J Cardiol. 1997; 29(6): 309-15.

**R14.** Verhoef P, Kok FJ, Kluijtmans LA, Blom HJ, Refsum H, Ueland PM, Kruyssen DA. The 677C-->T mutation in the methylenetetrahydrofolate reductase gene: associations withplasma total homocysteine levels and risk of coronary atherosclerotic disease. Atherosclerosis. 1997; 132(1): 105-13.

**R15.** Malinow MR, Nieto FJ, Kruger WD, Duell PB, Hess DL, Gluckman RA, Block PC, Holzgang CR, Anderson PH, Seltzer D, Upson B, Lin QR. The effects of folic acid supplementation on plasma total homocysteine are modulated by multivitamin use and methylenetetrahydrofolate reductase genotypes. Arterioscler Thromb Vasc Biol. 1997; 17(6): 1157-62.

**R16.** Christensen B, Frosst P, Lussier-Cacan S, Selhub J, Goyette P, Rosenblatt DS, Genest J Jr, Rozen R. Correlation of a common mutation in the methylenetetrahydrofolate reductase gene with plasma homocysteine in patients with premature coronary artery disease. Arterioscler Thromb Vasc Biol. 1997; 17(3): 569-73.

**R17.** Reinhardt D, Sigusch HH, Vogt SF, Farker K, Müller S, Hoffmann A. Absence of association between a common mutation in the methylenetetrahydrofolate reductase gene and the risk of coronary artery disease. Eur J Clin Invest. 1998; 28(1): 20-3.

**R18.** Girelli D, Friso S, Trabetti E, Olivieri O, Russo C, Pessotto R, Faccini G, Pignatti PF, Mazzucco A, Corrocher R. Methylenetetrahydrofolate reductase C677T mutation, plasma homocysteine, and folate insubjects from northern Italy with or without angiographically documented severe coronaryatherosclerotic disease: evidence for an important genetic-environmental interaction. Blood. 1998; 91(11): 4158-63.

**R19.** Ou T, Yamakawa-Kobayashi K, Arinami T, Amemiya H, Fujiwara H, Kawata K, Saito M, Kikuchi S, Noguchi Y, Sugishita Y, Hamaguchi H. Methylenetetrahydrofolate reductase and apolipoprotein E polymorphisms are independent riskfactors for coronary heart disease in Japanese: a case-control study. Atherosclerosis. 1998; 137(1): 23-8.

**R20.** Verhoef P, Rimm EB, Hunter DJ, Chen J, Willett WC, Kelsey K, Stampfer MJ. A common mutation in the methylenetetrahydrofolate reductase gene and risk of coronary heart disease: results among U.S. men. J Am Coll Cardiol. 1998; 32(2): 353-9.

**R21.** Malik NM, Syrris P, Schwartzman R, Kaski JC, Crossman DC, Francis SE, Carter ND, Jeffery S. Methylenetetrahydrofolate reductase polymorphism (C-677T) and coronary artery disease. Clin Sci (Lond). 1998; 95(3): 311-5.

**R22.** Verhoeff BJ, Trip MD, Prins MH, Kastelein JJ, Reitsma PH. The effect of a common methylenetetrahydrofolate reductase mutation on levels ofhomocysteine, folate, vitamin B12 and on the risk of premature atherosclerosis. Atherosclerosis. 1998; 141(1): 161-6.

**R23.** Todesco L, Angst C, Litynski P, Loehrer F, Fowler B, Haefeli WE. Methylenetetrahydrofolate reductase polymorphism, plasma homocysteine and age. Eur J Clin Invest. 1999; 29(12): 1003-9.

**R24.** Tsai MY, Welge BG, Hanson NQ, Bignell MK, Vessey J, Schwichtenberg K, Yang F, Bullemer FE, Rasmussen R, Graham KJ. Genetic causes of mild hyperhomocysteinemia in patients with premature occlusive coronary artery diseases. Atherosclerosis. 1999; 143(1): 163-70.

**R25.** Tokgözoğlu SL, Alikaşifoğlu M, Unsal, Atalar E, Aytemir K, Ozer N, Ovünç K, Usal O, Kes S, Tunçbilek E. Methylene tetrahydrofolate reductase genotype and the risk and extent of coronary artery diseasein a population with low plasma folate. Heart. 1999; 81(5): 518-22.

**R26.** Fernández-Arcás N, Dieguez-Lucena JL, Muñoz-Moran E, Ruiz-Galdón M, Espinosa-Caliani S, Aranda-Lara P, Martinez-Espigares S, Banderas-Donaire MJ, De Teresa-Galván E, Reyes-Engel A. The genotype interactions of methylenetetrahydrofolate reductase and renin-angiotensin systemgenes are associated with myocardial infarction. Atherosclerosis. 1999; 145(2): 293-300.

**R27.** Chao CL, Tsai HH, Lee CM, Hsu SM, Kao JT, Chien KL, Sung FC, Lee YT. The graded effect of hyperhomocysteinemia on the severity and extent of coronary atherosclerosis. Atherosclerosis. 1999; 147(2): 379-86.

**R28.** Ardissino D, Mannucci PM, Merlini PA, Duca F, Fetiveau R, Tagliabue L, Tubaro M, Galvani M, Ottani F, Ferrario M, Corral J, Margaglione M. Prothrombotic genetic risk factors in young survivors of myocardial infarction. Blood. 1999; 94(1): 46-51.

**R29.** Gardemann A, Weidemann H, Philipp M, Katz N, Tillmanns H, Hehrlein FW, Haberbosch W. The TT genotype of the methylenetetrahydrofolate reductase C677T gene polymorphism isassociated with the extent of coronary atherosclerosis in patients at high risk for coronary arterydisease. Eur Heart J. 1999; 20(8): 584-92.

**R30.** Mager A, Lalezari S, Shohat T, Birnbaum Y, Adler Y, Magal N, Shohat M. Methylenetetrahydrofolate reductase genotypes and early-onset coronary artery disease. Circulation. 1999; 100(24): 2406-10.

**R31.** Yoo JH, Park SC. Low plasma folate in combination with the 677 C-->T methylenetetrahydrofolate reductasepolymorphism is associated with increased risk of coronary artery disease in Koreans. Thromb Res. 2000; 97(2): 77-84.

**R32.** Zheng YZ, Tong J, Do XP, Pu XQ, Zhou BT. Prevalence of methylenetetrahydrofolate reductase C677T and its association with arterial andvenous thrombosis in the Chinese population. Br J Haematol. 2000; 109(4): 870-4.

**R33.** Chambers JC, Ireland H, Thompson E, Reilly P, Obeid OA, Refsum H, Ueland P, Lane DA, Kooner JS. Methylenetetrahydrofolate reductase 677 C-->T mutation and coronary heart disease risk in UK Indian Asians. Arterioscler Thromb Vasc Biol. 2000; 20(11): 2448-52.

**R34.** Virgos C, Joven J, Simó JM, Vilella E, Camps J, Arcelus R, Blanco-Vaca F, Figuera L, Martorell L. Homocyst(e)ine and the C677T mutation of methylenetetrahydrofolate reductase in survivors of premature myocardial infarction. Clin Biochem. 2000; 33(6): 509-12.

**R35.** Nakai K, Fusazaki T, Suzuki T, Ohsawa M, Ogiu N, Kamata J, Kawazoe K, Nakai K, Itoh C, Yanagisawa M, Ishida T, Hiramori K. Genetic polymorphism of 5,10-methylenetetrahydrofolate increases risk of myocardial infarctionand is correlated to elevated levels of homocysteine in the Japanese general population. Coron Artery Dis. 2000; 11(1): 47-51.

**R36.** Fowkes FG, Lee AJ, Hau CM, Cooke A, Connor JM, Lowe GD. Methylene tetrahydrofolate reductase (MTHFR) and nitric oxide synthase (ecNOS) genes andrisks of peripheral arterial disease and coronary heart disease: Edinburgh Artery Study. Atherosclerosis. 2000; 150(1): 179-85.

**R37.** Kawashiri M, Kajinami K, Nohara A, Yagi K, Inazu A, Koizumi J, Mabuchi H. Effect of common methylenetetrahydrofolate reductase gene mutation on coronary artery diseasein familial hypercholesterolemia. Am J Cardiol. 2000; 86(8): 840-5.

**R38.** Hong SH, Song J, Kim JQ. Genetic variation of the methylenetetrahydrofolate reductase and cystathionine beta-synthasegenes in Korean patients with coronary artery disease and a new polymorphism in intron 7. Mol Cell Probes. 2001; 15(2): 119-23.

**R39.** Thögersen AM, Nilsson TK, Dahlen G, Jansson JH, Boman K, Huhtasaari F, Hallmans G. Homozygosity for the C677-->T mutation of 5,10-methylenetetrahydrofolate reductase and totalplasma homocyst(e)ine are not associated with greater than normal risk of a first myocardial infarction in northern Sweden. Coron Artery Dis. 2001; 12(2): 85-90.

**R40.** Dilley A, Hooper WC, El-Jamil M, Renshaw M, Wenger NK, Evatt BL. Mutations in the genes regulating methylene tetrahydrofolate reductase (MTHFR C-->T677) and cystathione beta-synthase (CBS G-->A919, CBS T-->c833) are not associated with myocardial infarction in African Americans. Thromb Res. 2001; 103(2): 109-15.

**R41.** Kim CH, Hwang KY, Choi TM, Shin WY, Hong SY. The methylenetetrahydrofolate reductase gene polymorphism in Koreans with coronary artery disease. Int J Cardiol. 2001; 78(1): 13-7.

**R42.** Zhang G, Dai C. Gene polymorphisms of homocysteine metabolism-related enzymes in Chinese patients withocclusive coronary artery or cerebral vascular diseases. Thromb Res. 2001; 104(3): 187-95.

**R43.** Hanson NQ, Aras O, Yang F, Tsai MY. C677T and A1298C polymorphisms of the methylenetetrahydrofolate reductase gene: incidenceand effect of combined genotypes on plasma fasting and post-methionine load homocysteine invascular disease. Clin Chem. 2001; 47(4): 661-6.

**R44.** Gülec S, Aras O, Akar E, Tutar E, Omürlü K, Avci F, Dinçer I, Akar N, Oral D. Methylenetetrahydrofolate reductase gene polymorphism and risk of premature myocardial infarction. Clin Cardiol. 2001; 24(4): 281-4.

**R45.** Szczeklik A, Sanak M, Jankowski M, Dropiński J, Czachór R, Musiał J, Axenti I, Twardowska M, Brzostek T, Tendera M. Mutation A1298C of methylenetetrahydrofolate reductase: risk for early coronary disease notassociated with hyperhomocysteinemia. Am J Med Genet. 2001; 101(1): 36-9.

**R46.** Benes P, Kanková K, Muzík J, Groch L, Benedík J, Elbl L, Izakovicová-Hollá L, Vasků A, Znojil V, Vácha J. Methylenetetrahydrofolate reductase polymorphism, type II diabetes mellitus, coronary arterydisease, and essential hypertension in the Czech population. Mol Genet Metab. 2001; 73(2): 188-95.

**R47.** Meisel C, Cascorbi I, Gerloff T, Stangl V, Laule M, Müller JM, Wernecke KD, Baumann G, Roots I, Stangl K. Identification of six methylenetetrahydrofolate reductase(MTHFR) genotypes resulting fromcommon polymorphisms: impact on plasma homocysteine levels and development of coronaryartery disease. therosclerosis. 2001; 154(3): 651-8.

**R48.** Chen J, Zhang I, Cheng L, Li Y. The effect of polymorphisms of MTHER gene and vitamin B on hyperhomocysteinemia. J Tongji Med Univ. 2001; 21(1): 17-20.

**R49.** Raslová K, Smolková B, Vohnout B, Gasparovic J, Frohlich JJ. Risk factors for atherosclerosis in survivors of myocardial infarction and their spouses:comparison to controls without personal and family history of atherosclerosis. Metabolism. 2001; 50(1): 24-9.

**R50.** Pintó X, Vilaseca MA, Garcia-Giralt N, Ferrer I, Palá M, Meco JF, Mainou C, Ordovás JM, Grinberg D, Balcells S; Baix Llobregat Homocysteine Study Group. Homocysteine and the MTHFR 677C-->T allele in premature coronary artery disease. Case controland family studies. Eur J Clin Invest. 2001; 31(1): 24-30.

**R51.** Roest M, van der Schouw YT, Grobbee DE, Tempelman MJ, de Groot PG, Sixma JJ, Banga JD. Methylenetetrahydrofolate reductase 677 C/T genotype and cardiovascular disease mortality inpostmenopausal women. Am J Epidemiol. 2001; 153(7): 673-9.

**R52.** Hsu LA, Ko YL, Wang SM, Chang CJ, Hsu TS, Chiang CW, Lee YS. The C677T mutation of the methylenetetrahydrofolate reductase gene is not associated with the risk of coronary artery disease or venous thrombosis among Chinese in Taiwan. Hum Hered. 2001; 51(1-2): 41-5.

**R53.** Spiridonova MG, Stepanov VA, Puzyrev VP, Karpov RS. Analysis of gene complexes predisposing to coronary atherosclerosis. Genetika. 2002; 38(3): 383-92.

**R54.** Rothenbacher D, Fischer HG, Hoffmeister A, Hoffmann MM, März W, Bode G, Rosenthal J, Koenig W, Brenner H. Homocysteine and methylenetetrahydrofolate reductase genotype: association with risk of coronary heart disease and relation to inflammatory, hemostatic, and lipid parameters. Atherosclerosis. 2002; 162(1): 193-200.

**R55.** Mukherjee M, Joshi S, Bagadi S, Dalvi M, Rao A, Shetty KR. A low prevalence of the C677T mutation in the methylenetetrahydrofolate reductase gene in Asian Indians. Clin Genet. 2002; 61(2): 155-9.

**R56.** Friso S, Girelli D, Trabetti E, Stranieri C, Olivieri O, Tinazzi E, Martinelli N, Faccini G, Pignatti PF, Corrocher R. A1298C methylenetetrahydrofolate reductase mutation and coronary artery disease: relationshipswith C677T polymorphism and homocysteine/folate metabolism. Clin Exp Med. 2002; 2(1): 7-12.

**R57.** Blankenberg S, Rupprecht HJ, Peetz D, Bickel C, Hofman KP, Tiret L, Meyer J. Homocysteine, methylenetetrahydrofolate reductase/C677T genotype and risk for coronary heart disease. The AtheroGene study. Dtsch Med Wochenschr. 2002; 127(14): 729-35.

**R58.** Vasisht S, Gulati R, Narang R, Srivastava N, Srivastava LM, Manchanda SC, Agarwal DP. Polymorphism (C677T) in the 5,10-methylenetetrahydrofolate reductase (MTHFR) gene: Apreliminary study on north Indian men. Indian J Clin Biochem. 2002; 17(1): 99-107.

**R59.** Mao YM, Zhao FMi, Qin Q, Chen Q, Cheng JX, Xiao XQ, Cui RZ, Zhao BR. Assocoation of Methylenetetrahydrofolate Reductase Gene Polymorphism, Level of Homocysteine and Coronary Heart Disease. Tianjin Med J. 2002; 30(8): 451-453.

**R60.** Abu-Amero KK, Wyngaard CA, Dzimiri N. Prevalence and role of methylenetetrahydrofolate reductase 677 C-->T and 1298 A-->Cpolymorphisms in coronary artery disease in Arabs. Arch Pathol Lab Med. 2003; 127(10): 1349-52.

**R61.** Zak I, Niemiec P, Sarecka B, Balcerzyk A, Ciemniewski Z, Rudowska E, Dylag S. Carrier-state of D allele in ACE gene insertion/deletion polymorphism is associated with coronaryartery disease, in contrast to the C677-->T transition in the MTHFR gene. Acta Biochim Pol. 2003; 50(2): 527-34.

**R62.** Zuntar I, Topić E, Vukosavić D, Vuković V, Demarin V, Begonja A, Antoljak N, Simundić AM. Croatian population data for the C677T polymorphism in methylenetetrahydrofolate reductase:frequencies in healthy and atherosclerotic study groups. Clin Chim Acta. 2003; 335(1-2): 95-100.

**R63.** Brilakis ES, Berger PB, Ballman KV, Rozen R. Methylenetetrahydrofolate reductase (MTHFR) 677C>T and methionine synthase reductase(MTRR) 66A>G polymorphisms: association with serum homocysteine and angiographiccoronary artery disease in the era of flour products fortified with folic acid. Atherosclerosis. 2003; 168(2): 315-22.

**R64.** Girelli D, Martinelli N, Pizzolo F, Friso S, Olivieri O, Stranieri C, Trabetti E, Faccini G, Tinazzi E, Pignatti PF, Corrocher R. The interaction between MTHFR 677 C-->T genotype and folate status is a determinant of coronary atherosclerosis risk. J Nutr. 2003; 133(5): 1281-5.

**R65.** Botto N, Andreassi MG, Manfredi S, Masetti S, Cocci F, Colombo MG, Storti S, Rizza A, Biagini A. Genetic polymorphisms in folate and homocysteine metabolism as risk factors for DNA damage. Eur J Hum Genet. 2003; 11(9): 671-8.

**R66.** Atherosclerosis, Thrombosis, and Vascular Biology Italian Study Group. No evidence of association between prothrombotic gene polymorphisms and the development of acute myocardial infarction at a young age. Circulation. 2003; 107(8): 1117-22.

**R67.** Meleady R, Ueland PM, Blom H, Whitehead AS, Refsum H, Daly LE, Vollset SE, Donohue C, Giesendorf B, Graham IM, Ulvik A, Zhang Y, Bjorke Monsen AL; EC Concerted Action Project: Homocysteine and Vascular Disease. Thermolabile methylenetetrahydrofolate reductase, homocysteine, and cardiovascular diseaserisk: the European Concerted Action Project. Am J Clin Nutr. 2003; 77(1): 63-70.

**R68.** Ranjith N, Pegoraro RJ, Rom L. Risk factors and methylenetetrahydrofolate reductase gene polymorphisms in a young SouthAfrican Indian-based population with acute myocardial infarction. Cardiovasc J S Afr. 2003; 14(3): 127-32.

**R69.** Kalina A, Czeizel AE. The methylenetetrahydrofolate reductase gene polymorphism (C677T) is associated withincreased cardiovascular mortality in Hungary. Int J Cardiol. 2004; 97(2): 333-4.

**R70.** Tobin MD, Braund PS, Burton PR, Thompson JR, Steeds R, Channer K, Cheng S, Lindpaintner K, Samani NJ. Genotypes and haplotypes predisposing to myocardial infarction: a multilocus case-controlstudy. Eur Heart J. 2004; 25(6): 459-67.

**R71.** Kölling K, Ndrepepa G, Koch W, Braun S, Mehilli J, Schömig A, Kastrati A. Methylenetetrahydrofolate reductase gene C677T and A1298C polymorphisms, plasmahomocysteine, folate, and vitamin B12 levels and the extent of coronary artery disease. Am J Cardiol. 2004; 93(10): 1201-6.

**R72.** Almawi WY, Ameen G, Tamim H, Finan RR, Irani-Hakime N. Factor V G1691A, prothrombin G20210A, and methylenetetrahydrofolate reductase [MTHFR]C677T gene polymorphism in angiographically documented coronary artery disease. J Thromb Thrombolysis. 2004; 17(3): 199-205.

**R73.** McCarthy JJ, Parker A, Salem R, Moliterno DJ, Wang Q, Plow EF, Rao S, Shen G, Rogers WJ, Newby LK, Cannata R, Glatt K, Topol EJ; GeneQuest Investigators. Large scale association analysis for identification of genes underlying premature coronary heartdisease: cumulative perspective from analysis of 111 candidate genes. J Med Genet. 2004; 41(5): 334-41.

**R74.** Frederiksen J, Juul K, Grande P, Jensen GB, Schroeder TV, Tybjaerg-Hansen A, Nordestgaard BG. Methylenetetrahydrofolate reductase polymorphism (C677T), hyperhomocysteinemia, and risk of ischemic cardiovascular disease and venous thromboembolism: prospective and case-controlstudies from the Copenhagen City Heart Study. Blood. 2004; 104(10): 3046-51.

**R75.** Shioji K, Kokubo Y, Goto Y, Nonogi H, Iwai N. An association analysis between genetic polymorphisms of matrix metalloproteinase-3 and methylenetetrahydrofolate reductase and myocardial infarction in Japanese. J Thromb Haemost. 2004; 2(3): 527-8.

**R76.** Tanis BC, Blom HJ, Bloemenkamp DG, van den Bosch MA, Algra A, van der Graaf Y, Rosendaal FR. Folate, homocysteine levels, methylenetetrahydrofolate reductase (MTHFR) 677C --> T variant, and the risk of myocardial infarction in young women: effect of female hormones on homocysteine levels. J Thromb Haemost. 2004; 2(1): 35-41.

**R77.** Undas A, Jankowski M, Twardowska M, Padjas A, Jakubowski H, Szczeklik A. Antibodies to N-homocysteinylated albumin as a marker for early-onset coronary artery disease in men. Thromb Haemost. 2005; 93(2): 346-50.

**R78.** Sun J, Xu Y, Xue J, Zhu Y, Lu H. Methylenetetrahydrofolate reductase polymorphism associated with susceptibility to coronaryheart disease in Chinese type 2 diabetic patients. Mol Cell Endocrinol. 2005; 229(1-2): 95-101.

**R79.** Iqbal MP, Fatima T, Parveen S, Yousuf FA, Shafiq M, Mehboobali N, Khan AH, Azam I, Frossard PM. Lack of association of methylenetetrahydrofolate reductase 677C>T mutation with coronaryartery disease in a Pakistani population. J Mol Genet Med. 2005; 1(1): 26-32.

**R80.** Falchi A, Giovannoni L, Piras IS, Calo CM, Moral P, Vona G, Varesi L. Prevalence of genetic risk factors for coronary artery disease in Corsica island (France)Exp Mol Pathol. 2005; 79(3): 210-3.

**R81.** Yilmaz H, Isbir S, Agachan B, Ergen A, Farsak B, Isbir T. C677T mutation of methylenetetrahydrofolate reductase gene and serum homocysteine levels in Turkish patients with coronary artery disease. Cell Biochem Funct. 2006; 24(1): 87-90.

**R82.** Dalal AB, Tewari D, Tewari S, Sharma MK, Pradhan M, Gupta UR, Sinha N, Agarwal S. Association of coronary artery disease with polymorphisms of angiotensin-converting enzyme and methylenetetrahydrofolate reductase gene. Indian Heart J. 2006; 58(4): 330-5.

**R83.** Huh HJ, Chi HS, Shim EH, Jang S, Park CJ. Gene--nutrition interactions in coronary artery disease: correlation between the MTHFR C677Tpolymorphism and folate and homocysteine status in a Korean population. Thromb Res. 2006; 117(5): 501-6.

**R84.** Kerkeni M, Addad F, Chauffert M, Myara A, Gerhardt M, Chevenne D, Trivin F, Farhat MB, Miled A, Maaroufi K. Hyperhomocysteinaemia, methylenetetrahydrofolate reductase polymorphism and risk of coronary artery disease. Ann Clin Biochem. 2006; 43(Pt 3): 200-6.

**R85.** Rossi GP, Maiolino G, Seccia TM, Burlina A, Zavattiero S, Cesari M, Sticchi D, Pedon L, Zanchetta M, Pessina AC. Hyperhomocysteinemia predicts total and cardiovascular mortality in high-risk women. J Hypertens. 2006; 24(5): 851-9.

**R86.** Guerzoni AR, Pavarino-Bertelli EC, Godoy MF, Graça CR, Biselli PM, Souza DR, Bertollo EM. Methylenetetrahydrofolate reductase gene polymorphism and its association with coronary artery disease. Sao Paulo Med J. 2007; 125(1): 4-8.

**R87.** Bennouar N, Allami A, Azeddoug H, Bendris A, Laraqui A, El Jaffali A, El Kadiri N, Benzidia R, Benomar A, Fellat S, Benomar M. Thermolabile methylenetetrahydrofolate reductase C677T polymorphism and homocysteine arerisk factors for coronary artery disease in Moroccan population. J Biomed Biotechnol. 2007; 2007(1): 80687.

**R88.** Zhu HX, Ma XH, Huang TG, Ni YP, Zhou LJ. Relationship between methylenetetrahydrofolate reductase gene polymorphisms and coronary heart disease. PJCCPVD, 2007; 15: 8.

**R89.** Taymaz H, Erarslan S, Oner ET, Alkan T, Ağirbaşli M, Kirdar B. Sequence variations within the genes related to hemostatic imbalance and their impact oncoronary artery disease in Turkish population. Thromb Res. 2007; 119(1): 55-62.

**R90.** Lin PT, Huang MC, Lee BJ, Cheng CH, Tsai TP, Huang YC. High plasma homocysteine is associated with the risk of coronary artery disease independent of methylenetetrahydrofolate reductase 677C-->T genotypes. Asia Pac J Clin Nutr. 2008; 17(2): 330-8.

**R91.** Alam MA, Husain SA, Narang R, Chauhan SS, Kabra M, Vasisht S. Association of polymorphism in the thermolabile 5, 10-methylene tetrahydrofolate reductasegene and hyperhomocysteinemia with coronary artery disease. Mol Cell Biochem. 2008; 310(1-2): 111-7.

**R92.** Freitas AI, Mendonça I, Guerra G, Brión M, Reis RP, Carracedo A, Brehm A. Methylenetetrahydrofolate reductase gene, homocysteine and coronary artery disease: the A1298C polymorphism does matter. Inferences from a case study (Madeira, Portugal). Thromb Res. 2008; 122(5): 648-56.

**R93.** Szperl M, Dzielinska Z, Roszczynko M, Malek LA, Makowiecka-Ciesla M, Demkow M, Kadziela J, Prejbisz A, Florczak E, Zielinski T, Januszewicz A, Ruzyllo W. Genetic variants in hypertensive patients with coronary artery disease and coexistingatheromatous renal artery stenosis. Med Sci Monit. 2008; 14(12): CR611-6.

**R94.** Rassoul F, Richter V, Hentschel B, Geisel J, Herrmann W, Kuntze T. Plasma homocysteine levels & 677C-->T methylenetetrahydrofolate reductase genepolymorphism in patients with coronary artery disease of different severity. Indian J Med Res. 2008; 127(2): 154-8.

**R95.** Ilhan N, Kucuksu M, Kaman D, Ilhan N, Ozbay Y. The 677 C/T MTHFR polymorphism is associated with essential hypertension, coronary arterydisease, and higher homocysteine levels. Arch Med Res. 2008; 39(1): 125-30.

**R96.** Belkahla R, Omezzine A, Kchok K, Rebhi L, Ben Hadj Mbarek I, Rejeb J, Ben Rejeb N, Slimane N, Nabli N, Ben Abdelaziz A, Boughzala E, Bouslama A. Effect of polymorphisms on key enzymes in homocysteine metabolism, on plasmahomocysteine level and on coronary artery-disease risk in a Tunisian population. Ann Cardiol Angeiol (Paris). 2008; 57(4): 219-24.

**R97.** Rahimi Z, Nomani H, Mozafari H, Vaisi-Raygani A, Madani H, Malek-Khosravi S, Parsian A. Factor V G1691A, prothrombin G20210A and methylenetetrahydrofolate reductase polymorphism C677T are not associated with coronary artery disease and type 2 diabetes mellitus in western Iran. Blood Coagul Fibrinolysis. 2009; 20(4): 252-6.

**R98.** Vinukonda G, Shaik Mohammad N, Md Nurul Jain J, Prasad Chintakindi K, Rama Devi Akella R. Genetic and environmental influences on total plasma homocysteine and coronary artery disease(CAD) risk among South Indians. Clin Chim Acta. 2009; 405(1-2): 127-31.

**R99.** Ghazouani L, Abboud N, Mtiraoui N, Zammiti W, Addad F, Amin H, Almawi WY, Mahjoub T. Homocysteine and methylenetetrahydrofolate reductase C677T and A1298C polymorphisms in Tunisian patients with severe coronary artery disease. J Thromb Thrombolysis. 2009; 27(2): 191-7.

**R100.** Var A, Utük O, Akçali S, Sanlidağ T, Uyanik BS, Dinç G. Impact of hemostatic gene single point mutations in patients with non-diabetic coronary arterydisease. Mol Biol Rep. 2009; 36(8): 2235-43.

**R101.** Sabino A, Fernandes AP, Lima LM, Ribeiro DD, Sousa MO, de Castro Santos ME, Mota AP, Dusse LM, das Graças Carvalho M. Polymorphism in the methylenetetrahydrofolate reductase (C677T) gene and homocysteinelevels: a comparison in Brazilian patients with coronary arterial disease, ischemic stroke and peripheral arterial obstructive disease. J Thromb Thrombolysis. 2009; 27(1): 82-7.

**R102.** Tripathi R, Tewari S, Singh PK, Agarwal S. Association of homocysteine and methylene tetrahydrofolate reductase (MTHFR C677T) genepolymorphism with coronary artery disease (CAD) in the population of North India. Genet Mol Biol. 2010; 33(2): 224-8.

**R103.** Isordia-Salas I, Trejo-Aguilar A, Valadés-Mejía MG, Santiago-Germán D, Leaños-Miranda A, Mendoza-Valdéz L, Jáuregui-Aguilar R, Borrayo-Sánchez G,Majluf-Cruz A. C677T polymorphism of the 5,10 MTHFR gene in young Mexican subjects with ST-elevationmyocardial infarction. Arch Med Res. 2010; 41(4): 246-50.

**R104.** Dhar S, Chatterjee S, Ray S, Dutta A, Sengupta B, Chakrabarti S. Polymorphisms of methylenetetrahydrofolate reductase gene as the genetic predispositions of coronary artery diseases in eastern India. J Cardiovasc Dis Res. 2010; 1(3): 152-7.

**R105.** Vijaya Lakshmi SV, Naushad SM, Rupasree Y, Seshagiri Rao D, Kutala VK. Interactions of 5'-UTR thymidylate synthase polymorphism with 677C → T methylene tetrahydrofolate reductase and 66A → G methyltetrahydrofolate homocysteine methyl-transferase reductase polymorphisms determine susceptibility to coronary artery disease. J Atheroscler Thromb. 2011; 18(1): 56-64.

**R106.** Chen Q, Sun Y, Zhang L, Deng K, Xia H, Xing H, Xiang Y, Ran B, Zhang M, Xu X, Fu W. Detection of C677T mutation of MTHFR in subject with coronary heart disease by hairpin probewith enzymatic color on microarray. Biosens Bioelectron. 2011; 28(1): 84-90.

**R107.** Sarecka-Hujar B, Zak I, Krauze J. The TT genotype of the MTHFR 677C > T polymorphism increases susceptibility to premature coronary artery disease in interaction with some of the traditional risk factors. Acta Medica (Hradec Kralove). 2012; 55(4): 172-9.

**R108.** Gupta SK, Kotwal J, Kotwal A, Dhall A, Garg S. Role of homocysteine & MTHFR C677T gene polymorphism as risk factors for coronary artery disease in young Indians. Indian J Med Res. 2012; 135(4): 506-12.

**R109.** Andreassi MG, Adlerstein D, Carpeggiani C, Shehi E, Fantinato S, Ghezzi E, Botto N, Coceani M, L'abbate A. Individual and summed effects of high-risk genetic polymorphisms on recurrent cardiovascularevents following ischemic heart disease. Atherosclerosis. 2012; 223(2): 409-15.

**R110.** Balogh E, Bereczky Z, Katona E, Koszegi Z, Edes I, Muszbek L, Czuriga I. Interaction between homocysteine and lipoprotein(a) increases the prevalence of coronary arterydisease/myocardial infarction in women: a case-control study. Thromb Res. 2012; 129(2): 133-8.

**R111.** Trifonova EA, Spiridonova MG, Gabidulina TV, Urnov FD, Puzyrev VP, Stepanov VA. Analysis of the MTHFR gene linkage disequilibrium structure and association of polymorphicgene variants with coronary atherosclerosis. Genetika. 2012; 48(10): 1207-20.

**R112.** Kucukhuseyin O, Kurnaz O, Akadam-Teker AB, Isbir T, Bugra Z, Ozturk O, Yilmaz-Aydogan H. The association of MTHFR C677T gene variants and lipid profiles or body mass index in patients with diabetic and nondiabetic coronary heart disease. J Clin Lab Anal. 2013; 27(6): 427-34.

**R113.** Davis LA, Cannon GW, Pointer LF, Haverhals LM, Wolff RK, Mikuls TR, Reimold AM, Kerr GS, Richards JS, Johnson DS, Valuck R, Prochazka A, Caplan L. Cardiovascular events are not associated with MTHFR polymorphisms, but are associated with methotrexate use and traditional risk factors in US veterans with rheumatoid arthritis. J Rheumatol. 2013; 40(6): 809-17.

**R114.** Saffari B, Senemar S, Karimi M, Bahari M, Jooyan N, Yavarian M. An MTHFR variant, plasma homocysteine levels and late-onset coronary artery disease in subjects from southern Iran. Pak J Biol Sci. 2013; 16(16): 788-95.

**R115.** Senemar S, Saffari B, Sharifkazemi MB, Bahari M, Jooyan N, Dehaghani ED, Yavarian M. 5,10-methylene tetrahydrofolate reductase C677T gene polymorphism, homocysteineconcentration and the extent of premature coronary artery disease in southern Iran. EXCLI J. 2013; 12: 437-48.

**R116.** Tang O, Wu J, Qin F. Relationship between methylenetetrahydrofolate reductase gene polymorphism and the coronaryslow flow phenomenon. Coron Artery Dis. 2014; 25(8): 653-7.

**R117.** Yu X, Liu J, Zhu H, Xia Y, Gao L, Dong Y, Jia N, Shen W, Yang Y, Niu W. Synergistic association of DNA repair relevant gene polymorphisms with the risk of coronaryartery disease in northeastern Han Chinese. Thromb Res. 2014; 133(2): 229-34.

**R118.** Chen W, Hua K, Gu H, Zhang J, Wang L. Methylenetetrahydrofolate reductase C667T polymorphism is associated with increased risk of coronary artery disease in a Chinese population. Scand J Immunol. 2014; 80(5): 346-53.

**R119.** Ramkaran P, Phulukdaree A, Khan S, Moodley D, Chuturgoon AA. Methylenetetrahydrofolate reductase C677T polymorphism is associated with increased risk of coronary artery disease in young South African Indians. Gene. 2015; 571(1): 28-32.

**R120.** Tanguturi PR, Pullareddy B, Rama Krishna BS, Murthy DK. Lipoprotein lipase gene HindIII polymorphism and risk of myocardial infarction in South Indian population. Indian Heart J. 2013; 65(6): 653-7.

**R121.** Heidari MM, Khatami M, Hadadzadeh M, Kazemi M, Mahamed S, Malekzadeh P, Mirjalili M. Polymorphisms in NOS3, MTHFR, APOB and TNF-α Genes and Risk of Coronary AtheroscleroticLesions in Iranian Patients. Res Cardiovasc Med. 2015; 5(1): e29134.

**R122.** Lin X, Zhang W, Lu Q, Lei X, Wang T, Han X,N Ma A. Effect of MTHFR Gene Polymorphism Impact on Atherosclerosis via Genome-Wide Methylation. Med Sci Monit. 2016; 22: 341-5.

**R123.** Bickel C, Schnabel RB, Zengin E, Lubos E, Rupprecht H, Lackner K, Proust C, Tregouet D, Blankenberg S, Westermann D, Sinning C. Homocysteine concentration in coronary artery disease: Influence of three common singlenucleotide polymorphisms. Nutr Metab Cardiovasc Dis. 2017; 27(2): 168-175.

**R124.** Mazza A, Motti C, Nulli A, Marra G, Gnasso A, Pastore A, Federici G, Cortese C. Lack of association between carotid intima-media thickness and methylenetetrahydrofolate reductase gene polymorphism or serum homocysteine in non-insulin-dependent diabetes mellitus. Metabolism. 2000; 49(6): 718-23.

**R125.** Passaro A, Vanini A, Calzoni F, Alberti L, Zamboni PF, Fellin R, Solini A. Plasma homocysteine, methylenetetrahydrofolate reductase mutation and carotid damage inelderly healthy women. Atherosclerosis. 2001; 157(1): 175-80.

**R126.** Vulapalli R, Liang C, Zareba W, Moss AJ. Recurrent coronary events are not increased in postinfarction patients with methylenetetrahydrofolate reductase gene C677T polymorphism. Am J Cardiol. 2001; 87(11): 1289-92.

**R127.** Hu S, Gan PZ, Li J, Bi HM. The relationship between the mutat ion of methylenetetrahydrofolate reductase gene 677C→ T and the diabetic microangiopathy. Chin J Med Genet. 2001; 18(2): 118-121.

**R128.** Kawamoto R, Kohara K, Tabara Y, Miki T, Doi T, Tokunaga H, Konishi I. An association of 5,10-methylenetetrahydrofolate reductase (MTHFR) gene polymorphism andcommon carotid atherosclerosis. J Hum Genet. 2001; 46(9): 506-10.

**R129.** Mao YM, Zhao FMi, Qin Q, Chen Q, Cheng JX, Xiao XQ, Cui RZ, Zhao BR. Assocoation of Methylenetetrahydrofolate Reductase Gene Polymorphism, Level of Homocysteine and Coronary Heart Disease. Tianjin Med J. 2002; 30(8): 451-453.

**R130.** Butler R, Morris AD, Struthers AD. The T allele of the C(677)T 5,10-methylenetetrahydrofolate reductase (MTHFR) genepolymorphism may protect endothelial function in young, normal subjects. Arterioscler Thromb Vasc Biol. 2002; 22(1): 193-4.

**R131.** Tutuncu NB, Erbas T, Alikasifoglu M, Tuncbilek E. Thermolabile methylenetetrahydrofolate reductase enzyme genotype is frequent in type 2 diabetic patients with normal fasting homocysteine levels. J Intern Med. 2005; 257(5): 446-53.

**R132.** Pollex RL, Mamakeesick M, Zinman B, Harris SB, Hanley AJ, Hegele RA. Methylenetetrahydrofolate reductase polymorphism 677C>T is associated with peripheral arterialdisease in type 2 diabetes. Cardiovasc Diabetol. 2005; 4:17.

**R133.** Winkelmayer WC, Kramar R, Sunder-Plassmann G, Födinger M. Effects of single-nucleotide polymorphisms in MTHFR and MTRR on mortality and allograft lossin kidney transplant recipients. Kidney Int. 2005; 68(6): 2857-62.

**R134.** Koubaa N, Nakbi A, Smaoui M, Abid N, Chaaba R, Abid M, Hammami M. Hyperhomocysteinemia and elevated ox-LDL in Tunisian type 2 diabetic patients: role of geneticand dietary factors. Clin Biochem. 2007; 40(13-14): 1007-14.

**R135.** Pereira AC, Miyakawa AA, Lopes NH, Soares PR, de Oliveira SA, Cesar LA, Ramires JF, Hueb W, Krieger JE. Dynamic regulation of MTHFR mRNA expression and C677T genotype modulate mortality incoronary artery disease patients after revascularization. Thromb Res. 2007; 121(1): 25-32.

**R136.** Zee RY, Mora S, Cheng S, Erlich HA, Lindpaintner K, Rifai N, Buring JE, Ridker PM. Homocysteine, 5,10-methylenetetrahydrofolate reductase 677C>T polymorphism, nutrient intake, and incident cardiovascular disease in 24,968 initially healthy women. Clin Chem. 2007; 53(5): 845-51.

**R137.** Yao H, Ding LL, Wang XM, Xu FL. Polymorphisms of Methylenetetrahydrofolate Reductase C677T and Hyperuricemia in Males. Carcinogenesis; Teratogenesis & Mutagenesis. 2007; 19(1): 50-52.

**R138.** Maeda M, Yamamoto I, Fukuda M, Motomura T, Nishida M, Nonen S, Fujio Y, Kasayama S, Azuma J. MTHFR gene polymorphism is susceptible to diabetic retinopathy but not to diabetic nephropathyin Japanese type 2 diabetic patients. J Diabetes Complications. 2008; 22(2): 119-25.

**R139.** Huang L, Song XM, Zhu WL, Li Y. Plasma homocysteine and gene polymorphisms associated with the risk of hyperlipidemia innorthern Chinese subjects. Biomed Environ Sci. 2008; 21(6): 514-20.

**R140.** Collings A, Raitakari OT, Juonala M, Rontu R, Kähönen M, Hutri-Kähönen N, Rönnemaa T, Marniemi J, Viikari JS, Lehtimäki T. Associations of methylenetetrahydrofolate reductase C677T polymorphism with markers of subclinical atherosclerosis: the Cardiovascular Risk in Young Finns Study. Scand J Clin Lab Invest. 2008; 68(1): 22-30.

**R141.** Maitland-van der Zee AH, Lynch A, Boerwinkle E, Arnett DK, Davis BR, Leiendecker-Foster C, Ford CE, Eckfeldt JH. Interactions between the single nucleotide polymorphisms in the homocysteine pathway (MTHFR 677C>T, MTHFR 1298 A>C, and CBSins) and the efficacy of HMG-CoA reductase inhibitors in preventing cardiovascular disease in high-risk patients of hypertension: the GenHAT study. Pharmacogenet Genomics. 2008; 18(8): 651-6.

**R142.** Karadeniz M, Erdogan M, Zengi A, Eroglu Z, Tamsel S, Olukman M, Saygili F, Yilmaz C. Methylenetetrahydrofolate reductase C677T gene polymorphism in Turkish patients withpolycystic ovary syndrome. Endocrine. 2010; 38(1): 127-33.

**R143.** Chen AR, Zhang HG, Wang ZP, Fu SJ, Yang PQ, Ren JG, Ning YY, Hu XJ, Tian LH. C-reactive protein, vitamin B12 and C677T polymorphism of N-5,10-methylenetetrahydrofolatereductase gene are related to insulin resistance and risk factors for metabolic syndrome in Chinese population. Clin Invest Med. 2010; 33(5): E290-7.

**R144.** Iemitsu M, Murakami H, Sanada K, Yamamoto K, Kawano H, Gando Y, Miyachi M. Lack of carotid stiffening associated with MTHFR 677TT genotype in cardiorespiratory fit adults. Physiol Genomics. 2010; 42(2): 259-65.

**R145.** Zhang L, Yin RX, Liu WY, Miao L, Wu DF, Aung LH, Hu XJ, Cao XL, Wu JZ, Pan SL. Association of methylenetetrahydrofolate reductase C677T polymorphism and serum lipid levels in the Guangxi Bai Ku Yao and Han populations. Lipids Health Dis. 2010; 9:123.

**R146.** Lin L, Guo XZ, Ni Q, Gong YB, Wang B, Shi SH. Analysis on the Correlated Factors of Methylene Tetrahydrof olate Reductase C676T Mutation and the Different TCM Syndromes in Early Diabetic Nephropathy. Journal of Traditional Chinese Medicine. 2010; 51(5): 447-449.

**R147.** Miranda-Vilela AL, Lordelo GS, Akimoto AK, Alves PC, Pereira LC, Klautau-Guimarães Mde N, Grisolia CK. Genetic polymorphisms influence runners' responses to the dietary ingestion of antioxidantsupplementation based on pequi oil (Caryocar brasiliense Camb.): a before-after study. Genes Nutr. 2011; 6(4): 369-95.

**R148.** Siqueira ER, Oliveira CP, Muniz MT, Silva F, Pereira LM, Carrilho FJ. Methylenetetrahydrofolate reductase (MTHFR) C677T polymorphism and high plasmahomocysteine in chronic hepatitis C (CHC) infected patients from the Northeast of Brazil. Nutr J. 2011; 10:86.

**R149.** Taguchi T, Mori H, Hamada A, Yamori Y, Mori M. Serum folate, total homocysteine levels and methylenetetrahydrofolate reductase 677C>Tpolymorphism in young healthy female Japanese. Asia Pac J Clin Nutr. 2012; 21(2): 291-5.

**R150.** Yang Q, Bailey L, Clarke R, Flanders WD, Liu T, Yesupriya A, Khoury MJ, Friedman JM. Prospective study of methylenetetrahydrofolate reductase (MTHFR) variant C677T and risk of all-cause and cardiovascular disease mortality among 6000 US adults. Am J Clin Nutr. 2012; 95(5): 1245-53.

**R151.** Qin X, Li J, Cui Y, Liu Z, Zhao Z, Ge J, Guan D, Hu J, Wang Y, Zhang F, Xu X, Wang X, Xu X, Huo Y. MTHFR C677T and MTR A2756G polymorphisms and the homocysteine lowering efficacy ofdifferent doses of folic acid in hypertensive Chinese adults. Nutr J. 2012; 11:2.

**R152.** Jain M, Pandey P, Tiwary NK, Jain S. MTHFR C677T polymorphism is associated with hyperlipidemia in women with polycystic ovarysyndrome. J Hum Reprod Sci. 2012; 5(1): 52-6.

**R153.** Ford AH, Flicker L, Hankey GJ, Norman P, van Bockxmeer FM, Almeida OP. Homocysteine, methylenetetrahydrofolate reductase C677T polymorphism and cognitiveimpairment: the health in men study. Mol Psychiatry. 2012; 17(5): 559-66.

**R154.** Devlin AM, Ngai YF, Ronsley R, Panagiotopoulos C. Cardiometabolic risk and the MTHFR C677T variant in children treated with second-generationantipsychotics.Transl Psychiatry. 2012; 2: e71.

**R155.** Chmurzynska A, Malinowska AM, Twardowska-Rajewska J, Gawecki J. Elderly women: homocysteine reduction by short-term folic acid supplementation resulting inincreased glucose concentrations and affecting lipid metabolism (C677T MTHFR polymorphism). Nutrition. 2013; 29(6): 841-4.

**R156.** Lambrinoudaki I, Papadimitriou D, Kaparos G, Rizos D, Panoulis C, Deligeoroglou E, Alexandrou A, Auguolea A, Apostolakis M, Creatsa M, Kouskouni E. MTHFR C677T polymorphism modifies the effect of HRT on metabolic parameters inpostmenopausal women. Climacteric. 2013; 16(5): 568-75.

**R157.** Yin RX, Wu DF, Miao L, Htet Aung LH, Cao XL, Yan TT, Long XJ, Liu WY, Zhang L, Li M. Interactions of several single nucleotide polymorphisms and high body mass index on serum lipid traits. Biofactors. 2013; 39(3): 315-25.

**R158.** Bahadir A, Eroz R, Dikici S. Investigation of MTHFR C677T gene polymorphism, biochemical and clinical parameters in Turkish migraine patients: association with allodynia and fatigue. Cell Mol Neurobiol. 2013; 33(8): 1055-63.

**R159.** Yigit S, Karakus N, Inanir A. Association of MTHFR gene C677T mutation with diabetic peripheral neuropathy and diabetic retinopathy. Mol Vis. 2013; 19: 1626-30.

**R160.** Chen NY, Liu CW, Du LL, Xiao LP, Ge L, Wang YY, Wei Z, Wu HY, Luo CY, Liang L, Peng JH, Luo XQ, Yin RX, Nguyen CP, Pan SL. Enrichment of MTHFR 677 T in a Chinese long-lived cohort and its association with lipidmodulation. Lipids Health Dis. 2014; 13:104.

**R161.** Bahadır A,Eroz R, Türker Y. Does the MTHFR C677T gene polymorphism indicate cardiovascular disease risk in type 2 diabetes mellitus patients? Anatol J Cardiol. 2015; 15(7): 524-30.

**R162.** Asefi M, Vaisi-Raygani A, Khodarahmi R, Nemati H, Rahimi Z, Vaisi-Raygani H, Tavilani H, Pourmotabbed T. Methylentetrahydrofolatereductase (rs1801133) polymorphism and psoriasis: contribution tooxidative stress, lipid peroxidation and correlation with vascular adhesion protein 1, preliminaryreport. J Eur Acad Dermatol Venereol. 2014; 28(9): 1192-8.

**R163.** Jiang S, Zhao R, Pan M, Venners SA, Zhong G, Hsu YH. Associations of MTHFR and MTRR polymorphisms with serum lipid levels in Chinesehypertensive patients. Clin Appl Thromb Hemost. 2014; 20(4): 400-10.

**R164.** Lv CF, Zhang T, Li LL, Wang CY, Liu K. Correlation of metabolism-related indicators with MTHFR gene polymorphism in H type hypertension patients. China Tropical Medicine. 2014; 14(1): 86-88.

**R165.** Liang RL,Zhou YQ, Xie JM, LV WB, Kang B,Liang YQ, Chen YH, Li YX. Association of C677T gene polymorphisms of methylenetetrahydrofolate reductase and plasma homocysteine level wim hyperlipidemia. J South Med Univ. 2014; 34(8): 1195-1198.

**R166.** Husemoen LL, Skaaby T, Jørgensen T, Thuesen BH, Fenger M, Grarup N, Sandholt CH, Hansen T, Pedersen O, Linneberg A. MTHFR C677T genotype and cardiovascular risk in a general population without mandatory folicacid fortification. Eur J Nutr. 2014; 53(7): 1549-59.

**R167.** Li WX, Lv WW, Dai SX, Pan ML, Huang JF. Joint associations of folate, homocysteine and MTHFR, MTR and MTRRn gene polymorphismswith dyslipidemia in a Chinese hypertensive population: a cross-sectional study. Lipids Health Dis. 2015; 14: 101.

**R168.** Tetik Vardarlı A, Zengi A, Bozok Çetintaş V, Karadeniz M, Tamsel S, Küçükaslan AŞ, Köse T, Saygılı F, Eroglu Z. An Association Study Between Gene Polymorphisms of Folic Acid Metabolism Enzymes and Biochemical and Hormonal Parameters in Acromegaly. Genet Test Mol Biomarkers. 2015; 19(8): 431-8.

**R169.** Mohammadzadeh G, Karimi M, Bazyar M, Hosseini SM. Lack of association between MTHFR C677T polymorphism and breast cancer risk in Ahvaz, westsouth-Iran. Adv Biomed Res. 2016; 5:26.

**R170.** Chen SF, Zhou YL, Zhang YQ, Xia J, Chen YJ, Shan ZM, Hu QF. Relationship between serum folate, homocysteine and methylenetetrahydrofolate reductase gene polymorphism in elderly patients with cardiovascular and cerebrovascular disease. Zhejiang medical education. 2015; 14(1): 43-46.

**R171.** Morais CC, Alves MC, Augusto EM, Abdalla DS, Horst MA, Cominetti C. The MTHFR C677T Polymorphism Is Related to Plasma Concentration of Oxidized Low-Density Lipoprotein in Adolescents with Cardiovascular Risk Factors. J Nutrigenet Nutrigenomics. 2015; 8(3): 105-13.

**R172.** Zhi X, Yang B, Fan S, Wang Y, Wei J, Zheng Q, Sun G. Gender-specific interactions of MTHFR C677T and MTRR A66G polymorphisms withoverweight/obesity on serum lipid levels in a Chinese Han population. Lipids Health Dis. 2016; 15(1): 185.

**R173.** Ghogomu SM, Ngolle NE, Mouliom RN, Asa BF. Association between the MTHFR C677T gene polymorphism and essential hypertension in SouthWest Cameroon. Genet Mol Res. 2016; 15(1).

**R174.** El Hajj Chehadeh SW, Jelinek HF, Al Mahmeed WA, Tay GK, Odama UO, Elghazali GE, Al Safar HS. Relationship between MTHFR C677T and A1298C gene polymorphisms and complications of type2 diabetes mellitus in an Emirati population. Meta Gene. 2016; 9: 70-5.

**R175.** Abd-Elmawla MA, Rizk SM, Youssry I, Shaheen AA. Impact of Genetic Polymorphism of methylenetetrahydrofolate reductase C677T on Development of Hyperhomocysteinemia and Related Oxidative Changes in Egyptian β-Thalassemia MajorPatients. PLoS One. 2016; 11(5): e0155070.

**R176.** Rashed L, Abdel Hay R, AlKaffas M, Ali S, Kadry D, Abdallah S. Studying the association between methylenetetrahydrofolate reductase (MTHFR) 677 gene polymorphism, cardiovascular risk and lichen planus. J Oral Pathol Med. 2017; 46(10): 1023-1029.

**R177.** Fan GJ, Xu R, Zhang Q, Yun L, Cao YL, Zhang CM. Correlation between methylenetetrahydrofolate reductase gene C677T polymorphism and blood lipid abnormality among hypertensives. Chin J Arterioscler. 2017; 25(2): 153-158.

**R178.** Shang GY, Wang HY, Liu JB, Yuan P, Zhou YY, Li LH. methylenetetrahydrofolate reductase gene C677T polymorphism and lower extremity vascular disease in patients with type 2 diabetes mellitus. Chin J Health Care Med. 2017; 19(3): 257-258.

**R179.** Abd El-Aziz TA, Mohamed RH. Influence of MTHFR C677T gene polymorphism in the development of cardiovascular disease in Egyptian patients with rheumatoid arthritis. Gene. 2017; 610: 127-132.
