# Supplementary material for: Associations of the MTHFR rs1801133 polymorphism with coronary artery disease and lipid levels: a systematic review and updated meta-analysis
Source: Lipids Health Dis. 2018 Aug 17;17:191. doi: 10.1186/s12944-018-0837-y (PMC6097444; doi:10.1186/s12944-018-0837-y)
Supplement: Supplementary file 2 — Table S1. Characteristics of the individual studies included in the meta-analysis between the MTHFR rs1801133 polymorphism and CAD; Table S2. Characteristics of the individual studies included in the meta-analysis between the MTHFR rs1801133 polymorphism and plasma lipid levels; Table S3. Plasma lipid levels according to the genotypes of the MTHFR rs1801133 polymorphism. (DOC 544 kb) [file 12944_2018_837_MOESM2_ESM.doc]

**Table S1.** Characteristics of the individual studies included in the meta-analysis between the *MTHFR* rs1801133 polymorphism and CAD

| **First author** | **Year** | **Gender** | **Ethnicity** | **Type of study** | **Genotyping method** | **Sample size**  **(case/control)** | **Case (N)** | | |  | **Control (N)** | | | **MAF**  **(control)** | ***P*_HWE_** |
| --- | --- | --- | --- | --- | --- | --- | --- | --- | --- | --- | --- | --- | --- | --- | --- |
|  |  |  |  |  |  |  | **CC** | **CT** | **TT** |  | **CC** | **CT** | **TT** |  |  |
| Wilcken DE [R1] | 1996 | M/F | Caucasian | Case/Control | PCR-RFLP | 456/225 | 186 | 217 | 53 |  | 88 | 113 | 24 | 0.36 | 0.16 |
| Ma J [R2] | 1996 | M | Caucasian | Nested Case/Control | PCR-RFLP | 293/290 | 136 | 124 | 33 |  | 135 | 116 | 39 | 0.33 | 0.08 |
| Adams M [R3] | 1996 | M/F | Caucasian | Nested Case/Control | PCR-RFLP | 310/222 | 133 | 145 | 32 |  | 96 | 97 | 29 | 0.35 | 0.57 |
| Schmitz C [R4] | 1996 | M/F | Caucasian | Nested Case/Control | PCR-RFLP | 190/188 | 95 | 66 | 29 |  | 71 | 90 | 27 | 0.38 | 0.86 |
| Izumi M [R5] | 1996 | M/F | Asian | Case/Control | PCR-RFLP | 250/201 | 90 | 110 | 50 |  | 74 | 102 | 25 | 0.38 | 0.26 |
| Gallagher PM [R6] | 1996 | M/F | Caucasian | Case/Control | PCR-RFLP | 111/105 | 44 | 48 | 19 |  | 53 | 45 | 7 | 0.28 | 0.53 |
| Brulhart MC [R7] | 1997 | M/F | Caucasian | Case/Control | PCR-RFLP | 193/456 | 86 | 84 | 23 |  | 188 | 195 | 73 | 0.37 | 0.06 |
| Schwartz SM [R8] | 1997 | F | Caucasian | Case/Control | PCR-RFLP | 69/338 | 28 | 34 | 7 |  | 154 | 141 | 43 | 0.34 | 0.23 |
| Brugada R [R9] | 1997 | M/F | Caucasian | Case/Control | PCR-RFLP | 234/231 | 117 | 101 | 16 |  | 105 | 110 | 16 | 0.31 | 0.07 |
| van Bockxmeer FM [R10] | 1997 | M/F | Caucasian | Case/Control | PCR-RFLP | 139/73 | 61 | 63 | 15 |  | 35 | 30 | 8 | 0.32 | 0.68 |
| Anderson JL [R11] | 1997 | M/F | Caucasian | Case/Control | PCR-RFLP | 730/722 | 331 | 299 | 80 |  | 330 | 311 | 81 | 0.33 | 0.55 |
| Kluijtmans LAJ [R12] | 1997 | M | Caucasian | Case/Control | PCR-RFLP | 735/1250 | 337 | 328 | 70 |  | 617 | 527 | 106 | 0.30 | 0.66 |
| Morita H [R13] | 1997 | M | Asian | Case/Control | PCR-RFLP | 362/778 | 117 | 188 | 57 |  | 338 | 361 | 79 | 0.33 | 0.22 |
| Verhoef P [R14] | 1997 | M/F | Caucasian | Case/Control | PCR-RFLP | 131/100 | 59 | 59 | 13 |  | 45 | 48 | 7 | 0.31 | 0.22 |
| Malinow MR [R15] | 1997 | M/F | Caucasian | Case/Control | PCR-RFLP | 140/102 | 40 | 83 | 17 |  | 49 | 45 | 8 | 0.30 | 0.60 |
| Christensen B [R16] | 1997 | M/F | Caucasian | Case/Control | PCR-RFLP | 152/121 | 62 | 68 | 22 |  | 47 | 61 | 13 | 0.36 | 0.30 |
| Reinhardt D [R17] | 1998 | M/F | Caucasian | Case/Control | PCR-RFLP | 180/104 | 91 | 66 | 23 |  | 49 | 46 | 9 | 0.31 | 0.70 |
| Girelli D [R18] | 1998 | M/F | Caucasian | Case/Control | PCR-RFLP | 278/137 | 90 | 148 | 40 |  | 42 | 70 | 25 | 0.44 | 0.66 |
| Ou T [R19] | 1998 | M/F | Asian | Case/Control | PCR-RFLP | 214/310 | 69 | 84 | 61 |  | 110 | 158 | 42 | 0.39 | 0.21 |
| Verhoef P [R20] | 1998 | M | Caucasian | Nested Case/Control | PCR-RFLP | 500/500 | 230 | 209 | 61 |  | 228 | 200 | 72 | 0.34 | 0.01 |
| Malik NM [R21] | 1998 | M/F | Caucasian | Case/Control | PCR-RFLP | 267/233 | 107 | 134 | 26 |  | 106 | 110 | 17 | 0.31 | 0.11 |
| Verhoeff BJ [R22] | 1998 | M/F | Caucasian | Case/Control | PCR-RFLP | 257/272 | 137 | 93 | 27 |  | 129 | 105 | 38 | 0.33 | 0.03 |
| Todesco L [R23] | 1999 | M/F | Caucasian | Case/Control | PCR-RFLP | 75/224 | 30 | 34 | 11 |  | 103 | 93 | 28 | 0.33 | 0.33 |
| Tsai MY [R24] | 1999 | M/F | Caucasian | Case/Control | PCR-RFLP | 376/82 | 159 | 177 | 40 |  | 35 | 35 | 12 | 0.36 | 0.51 |
| Tokgözoğlu SL [R25] | 1999 | M/F | Other ethnicity | Case/Control | PCR-RFLP | 151/91 | 69 | 71 | 11 |  | 47 | 39 | 5 | 0.27 | 0.40 |
| Fernández-Arcás N [R26] | 1999 | M/F | Caucasian | Case/Control | PCR-RFLP | 221/198 | 85 | 83 | 53 |  | 82 | 76 | 40 | 0.39 | 0.01 |
| Chao CL [R27] | 1999 | M/F | Asian | Case/Control | PCR-RFLP | 116/76 | 60 | 47 | 9 |  | 41 | 25 | 10 | 0.30 | 0.07 |
| Ardissino D [R28] | 1999 | M/F | Caucasian | Case/Control | PCR-RFLP | 200/200 | 68 | 97 | 35 |  | 60 | 102 | 38 | 0.45 | 0.65 |
| Gardemann A [R29] | 1999 | M | Caucasian | Case/Control | Not clear | 1893/560 | 891 | 805 | 197 |  | 242 | 254 | 64 | 0.34 | 0.83 |
| Mager A [R30] | 1999 | M/F | Caucasian | Case/Control | PCR-RFLP | 169/313 | 52 | 85 | 32 |  | 130 | 139 | 44 | 0.36 | 0.49 |
| Yoo JH [R31] | 2000 | M | Asian | Case/Control | PCR-RFLP | 187/122 | 50 | 115 | 22 |  | 42 | 64 | 16 | 0.39 | 0.27 |
| Zheng YZ [R32] | 2000 | M/F | Asian | Case/Control | PCR-RFLP | 100/122 | 54 | 41 | 5 |  | 62 | 45 | 15 | 0.31 | 0.14 |
| Chambers JC [R33] | 2000 | M | Asian | Case/Control | PCR-RFLP | 454/805 | 268 | 152 | 34 |  | 467 | 285 | 53 | 0.24 | 0.29 |
| Virgos C [R34] | 2000 | M | Caucasian | Case/Control | PCR-RFLP | 72/72 | 34 | 33 | 5 |  | 27 | 31 | 14 | 0.41 | 0.35 |
| Nakai K [R35] | 2000 | M | Asian | Case/Control | PCR-RFLP | 230/198 | 93 | 95 | 42 |  | 81 | 96 | 21 | 0.35 | 0.34 |
| Fowkes FG [R36] | 2000 | M/F | Caucasian | Nested Case/Control | PCR-RFLP | 137/300 | 59 | 66 | 12 |  | 132 | 147 | 21 | 0.32 | 0.02 |
| Kawashiri M [R37] | 2000 | M | Asian | Case/Control | PCR-RFLP | 99/100 | 33 | 51 | 15 |  | 48 | 45 | 7 | 0.30 | 0.41 |
| Hong SH [R38] | 2001 | M/F | Asian | Case/Control | PCR-RFLP | 140/140 | 40 | 74 | 26 |  | 37 | 78 | 25 | 0.46 | 0.15 |
| Thögersen AM [R39] | 2001 | M/F | Caucasian | Nested Case/Control | PCR-RFLP | 69/129 | 32 | 32 | 5 |  | 71 | 51 | 7 | 0.25 | 0.58 |
| Dilley A [40] | 2001 | M/F | Caucasian | Case/Control | PCR-RFLP | 110/185 | 91 | 17 | 2 |  | 153 | 28 | 4 | 0.10 | 0.06 |
| Kim C-H [41] | 2001 | M/F | Asian | Case/Control | Not clear | 85/152 | 30 | 41 | 14 |  | 56 | 68 | 28 | 0.41 | 0.36 |
| Zhang G [42] | 2001 | M/F | Asian | Case/Control | PCR-RFLP | 73/100 | 32 | 33 | 8 |  | 37 | 47 | 16 | 0.40 | 0.87 |
| Hanson NQ [R43] | 2001 | M/F | Caucasian | Case/Control | PCR-RFLP | 772/329 | 324 | 364 | 84 |  | 130 | 158 | 41 | 0.36 | 0.51 |
| Gülec S [R44] | 2001 | M | Other ethnicity | Case/Control | PCR-RFLP | 96/100 | 42 | 39 | 15 |  | 60 | 35 | 5 | 0.23 | 0.97 |
| Szczeklik A [R45] | 2001 | M | Caucasian | Case/Control | PCR-RFLP | 161/211 | 105 | 39 | 17 |  | 118 | 75 | 18 | 0.26 | 0.23 |
| Benes P [R46] | 2001 | M/F | Caucasian | Case/Control | PCR-RFLP | 641/209 | 264 | 309 | 68 |  | 86 | 106 | 17 | 0.33 | 0.05 |
| Meisel C [R47] | 2001 | M/F | Caucasian | Case/Control | PCR-RFLP | 981/981 | 458 | 442 | 81 |  | 443 | 442 | 96 | 0.32 | 0.35 |
| Chen J [R48] | 2001 | M/F | Asian | Case/Control | PCR-RFLP | 51/30 | 22 | 23 | 6 |  | 9 | 16 | 5 | 0.43 | 0.64 |
| Raslová K [R49] | 2001 | M/F | Caucasian | Case/Control | PCR-RFLP | 58/69 | 22 | 29 | 7 |  | 36 | 29 | 4 | 0.27 | 0.56 |
| Pintó X [R50] | 2001 | M | Caucasian | Case/Control | PCR-RFLP | 76/95 | 31 | 34 | 11 |  | 39 | 43 | 13 | 0.36 | 0.83 |
| Roest M [51] | 2001 | F | Caucasian | Nested Case/Control | Real-time PCR assay | 501/515 | 235 | 220 | 46 |  | 209 | 248 | 58 | 0.35 | 0.22 |
| Hsu LA [52] | 2001 | M/F | Asian | Case/Control | PCR-RFLP | 218/218 | 120 | 85 | 13 |  | 125 | 78 | 15 | 0.25 | 0.56 |
| Spiridonova MG [R53] | 2002 | M/F | Caucasian | Case/Control | PCR-RFLP | 94/122 | 44 | 41 | 9 |  | 64 | 48 | 10 | 0.28 | 0.81 |
| Rothenbacher D [R54] | 2002 | M/F | Caucasian | Case/Control | PCR-RFLP | 312/479 | 155 | 126 | 31 |  | 219 | 210 | 50 | 0.32 | 0.97 |
| Mukherjee M [R55] | 2002 | M/F | Asian | Case/Control | PCR-RFLP | 251/205 | 155 | 96 | 0 |  | 137 | 63 | 5 | 0.18 | 0.47 |
| Friso S [R56] | 2002 | M/F | Caucasian | Case/Control | PCR-RFLP | 302/168 | 100 | 156 | 46 |  | 48 | 90 | 30 | 0.45 | 0.28 |
| Blankenberg S [R57] | 2002 | M/F | Caucasian | Case/Control | PCR-RFLP | 949/343 | 406 | 439 | 104 |  | 148 | 155 | 40 | 0.34 | 0.95 |
| Vasisht S [R58] | 2002 | M | Asian | Case/Control | PCR-RFLP | 141/182 | 98 | 32 | 11 |  | 39 | 141 | 2 | 0.40 | 0.00 |
| Mao Y [R59] | 2002 | M/F | Asian | Case/Control | PCR-RFLP | 298/136 | 53 | 142 | 103 |  | 27 | 61 | 48 | 0.58 | 0.34 |
| Abu-Amero KK [R60] | 2003 | M/F | Caucasian | Case/Control | PCR-RFLP | 545/625 | 350 | 175 | 20 |  | 451 | 161 | 13 | 0.15 | 0.76 |
| Zak I [R61] | 2003 | M/F | Caucasian | Case/Control | PCR-RFLP | 66/111 | 29 | 31 | 6 |  | 47 | 56 | 8 | 0.32 | 0.11 |
| Zuntar I [R62] | 2003 | M/F | Caucasian | Case/Control | PCR-RFLP | 247/298 | 114 | 111 | 22 |  | 134 | 146 | 18 | 0.31 | 0.01 |
| Brilakis ES [R63] | 2003 | M/F | Caucasian | Case/Control | PCR-RFLP | 382/122 | 173 | 172 | 37 |  | 53 | 52 | 17 | 0.35 | 0.46 |
| Girelli D [R64] | 2003 | M/F | African | Case/Control | PCR-RFLP | 433/222 | 146 | 217 | 70 |  | 75 | 105 | 42 | 0.43 | 0.63 |
| Botto N [R65] | 2003 | M/F | Caucasian | Case/Control | PCR-RFLP | 51/17 | 14 | 25 | 12 |  | 5 | 10 | 2 | 0.41 | 0.38 |
| Atherosclerosis, Thrombosis, and Vascular Biology Italian Study Group [R66] | 2003 | M/F | Caucasian | Case/Control | PCR-RFLP | 1209/1235 | 371 | 547 | 291 |  | 363 | 620 | 252 | 0.46 | 0.67 |
| Meleady R [R67] | 2003 | M/F | Caucasian | Case/Control | PCR-RFLP | 711/747 | 313 | 307 | 91 |  | 352 | 314 | 81 | 0.32 | 0.38 |
| Ranjith N [R68] | 2003 | M/F | African | Case/Control | PCR-RFLP | 195/300 | 166 | 29 | 0 |  | 238 | 58 | 4 | 0.11 | 0.83 |
| Kalina A [R69] | 2004 | M/F | Caucasian | Case/Control | PCR-RFLP | 315/96 | 138 | 146 | 31 |  | 44 | 40 | 12 | 0.33 | 0.54 |
| Tobin MD [R70] | 2004 | M/F | Caucasian | Case/Control | PCR-RFLP | 547/505 | 236 | 246 | 65 |  | 214 | 230 | 61 | 0.35 | 0.95 |
| Kölling K [R71] | 2004 | M/F | Caucasian | Case/Control | Real-time PCR assay | 2121/617 | 915 | 955 | 251 |  | 266 | 283 | 68 | 0.34 | 0.57 |
| Almawi WY [R72] | 2004 | M/F | Other ethnicity | Case/Control | PCR-RFLP | 96/404 | 27 | 39 | 30 |  | 220 | 166 | 18 | 0.25 | 0.05 |
| McCarthy JJ [R73] | 2004 | M/F | Caucasian | Case/Control | Real-time PCR assay | 482/818 | 224 | 180 | 78 |  | 382 | 344 | 92 | 0.32 | 0.28 |
| Frederiksen J [R74] | 2004 | M/F | Caucasian | Case/Control | PCR-RFLP | 3510/15253 | 3212 | 287 | 11 |  | 14006 | 1217 | 30 | 0.04 | 0.51 |
| Shioji K [R75] | 2004 | F | Asian | Case/Control | Real-time PCR assay | 70/1001 | 33 | 24 | 13 |  | 370 | 467 | 164 | 0.40 | 0.42 |
| Tanis BC [R76] | 2004 | F | Caucasian | Case/Control | PCR-RFLP | 181/601 | 78 | 81 | 22 |  | 280 | 262 | 59 | 0.32 | 0.84 |
| Undas A [R77] | 2005 | M | Caucasian | Case/Control | PCR-RFLP | 88/100 | 57 | 22 | 9 |  | 57 | 35 | 8 | 0.26 | 0.43 |
| Sun J [R78] | 2005 | M/F | Asian | Case/Control | PCR-RFLP | 126/102 | 43 | 52 | 31 |  | 58 | 26 | 18 | 0.30 | 0.00 |
| Iqbal MP [R79] | 2005 | M/F | Caucasian | Case/Control | PCR-RFLP | 397/225 | 279 | 110 | 8 |  | 161 | 57 | 7 | 0.16 | 0.48 |
| Falchi A [R80] | 2005 | M/F | Caucasian | Case/Control | PCR-RFLP | 100/100 | 37 | 43 | 20 |  | 42 | 54 | 4 | 0.31 | 0.01 |
| Yilmaz H [R81] | 2006 | M/F | Other ethnicity | Case/Control | PCR-RFLP | 79/93 | 40 | 32 | 7 |  | 43 | 44 | 6 | 0.30 | 0.23 |
| Dalal AB [R82] | 2006 | M/F | Asian | Case/Control | PCR-RFLP | 268/90 | 207 | 52 | 9 |  | 69 | 15 | 6 | 0.15 | 0.00 |
| Huh HJ [R83] | 2006 | M/F | Asian | Case/Control | Real-time PCR assay | 163/50 | 57 | 63 | 43 |  | 18 | 23 | 9 | 0.41 | 0.73 |
| Kerkeni M [R84] | 2006 | M/F | African | Case/Control | PCR-RFLP | 100/120 | 49 | 35 | 16 |  | 58 | 55 | 7 | 0.29 | 0.19 |
| Rossi GP [R85] | 2006 | F | Caucasian | Case/Control | Real-time PCR assay | 100/100 | 34 | 43 | 23 |  | 32 | 45 | 23 | 0.46 | 0.35 |
| Guerzoni AR [R86] | 2007 | M/F | Caucasian | Case/Control | PCR-RFLP | 91/36 | 26 | 59 | 6 |  | 14 | 20 | 2 | 0.33 | 0.13 |
| Bennouar N [R87] | 2007 | M/F | Other ethnicity | Case/Control | PCR-RFLP | 210/190 | 101 | 78 | 31 |  | 113 | 61 | 16 | 0.24 | 0.07 |
| Zhu H [R88] | 2007 | M/F | Asian | Case/Control | PCR-RFLP | 50/50 | 13 | 22 | 15 |  | 26 | 14 | 10 | 0.34 | 0.01 |
| Taymaz H [R89] | 2007 | M/F | Other ethnicity | Case/Control | PCR-RFLP | 133/41 | 78 | 41 | 14 |  | 22 | 17 | 2 | 0.26 | 0.57 |
| Lin PT [R90] | 2008 | M/F | Asian | Case/Control | PCR-RFLP | 121/155 | 66 | 47 | 8 |  | 88 | 57 | 10 | 0.25 | 0.85 |
| Alam MA [R91] | 2008 | M/F | Asian | Case/Control | PCR-RFLP | 84/100 | 57 | 23 | 4 |  | 80 | 19 | 1 | 0.11 | 0.91 |
| Freitas AI [R92] | 2008 | M/F | Caucasian | Case/Control | PCR-RFLP | 298/510 | 130 | 136 | 32 |  | 262 | 200 | 48 | 0.29 | 0.28 |
| Szperl M [R93] | 2008 | M/F | Caucasian | Case/Control | PCR-RFLP | 151/195 | 73 | 65 | 13 |  | 75 | 108 | 12 | 0.34 | 0.00 |
| Rassoul F [R94] | 2008 | M | Caucasian | Case/Control | PCR-RFLP | 112/60 | 46 | 47 | 19 |  | 32 | 21 | 7 | 0.29 | 0.24 |
| Ilhan N [R95] | 2008 | M/F | Other ethnicity | Case/Control | Real-time PCR assay | 100/100 | 52 | 44 | 4 |  | 72 | 26 | 2 | 0.15 | 0.84 |
| Belkahla R [R96] | 2008 | M/F | African | Case/Control | PCR-RFLP | 173/78 | 88 | 30 | 55 |  | 51 | 19 | 8 | 0.22 | 0.01 |
| Rahimi Z [R97] | 2009 | M/F | Caucasian | Case/Control | PCR-RFLP | 117/59 | 68 | 40 | 9 |  | 33 | 22 | 4 | 0.25 | 0.90 |
| Vinukonda G [R98] | 2009 | M/F | Asian | Case/Control | PCR-RFLP | 108/108 | 75 | 31 | 2 |  | 88 | 20 | 0 | 0.09 | 0.29 |
| Ghazouani L [R99] | 2009 | M/F | African | Case/Control | PCR-RFLP | 352/390 | 157 | 149 | 46 |  | 247 | 123 | 20 | 0.21 | 0.36 |
| Var A [R100] | 2009 | M/F | Other ethnicity | Case/Control | Real-time PCR assay | 86/91 | 58 | 24 | 4 |  | 52 | 23 | 16 | 0.30 | 0.00 |
| Sabino A [R101] | 2009 | M/F | Other ethnicity | Case/Control | PCR-RFLP | 43/37 | 23 | 18 | 2 |  | 24 | 12 | 1 | 0.19 | 0.73 |
| Tripathi R [R102] | 2010 | M/F | Asian | Case/Control | PCR-RFLP | 329/331 | 260 | 60 | 9 |  | 288 | 38 | 5 | 0.07 | 0.01 |
| Isordia-Salas I [R103] | 2010 | M/F | Other ethnicity | Case/Control | PCR-RFLP | 167/167 | 38 | 75 | 54 |  | 42 | 78 | 47 | 0.51 | 0.40 |
| Dhar S [R104] | 2010 | M/F | Asian | Case/Control | PCR-RFLP | 217/255 | 112 | 47 | 58 |  | 186 | 36 | 33 | 0.20 | 0.00 |
| Vijaya Lakshmi SV [R105] | 2011 | M/F | Asian | Case/Control | PCR-RFLP | 350/280 | 256 | 88 | 6 |  | 231 | 49 | 0 | 0.09 | 0.11 |
| Chen Q [R106] | 2011 | M/F | Asian | Case/Control | Real-time PCR assay | 98/59 | 31 | 56 | 11 |  | 39 | 18 | 2 | 0.19 | 0.97 |
| Sarecka-Hujar B [R107] | 2012 | M/F | Caucasian | Case/Control | PCR-RFLP | 152/121 | 65 | 72 | 15 |  | 59 | 56 | 6 | 0.28 | 0.11 |
| Gupta SK [R108] | 2012 | M/F | Asian | Case/Control | PCR-RFLP | 199/200 | 132 | 64 | 3 |  | 154 | 45 | 1 | 0.12 | 0.23 |
| Andreassi MG [R109] | 2012 | M/F | Caucasian | Nested Case/Control | Real-time PCR assay | 119/379 | 32 | 46 | 41 |  | 114 | 186 | 79 | 0.45 | 0.85 |
| Balogh E [R110] | 2012 | F | Caucasian | Case/Control | Real-time PCR assay | 653/906 | 276 | 312 | 65 |  | 411 | 399 | 96 | 0.33 | 0.95 |
| Trifonova EA [R111] | 2012 | M/F | Caucasian | Case/Control | PCR-RFLP | 100/100 | 52 | 40 | 8 |  | 55 | 39 | 6 | 0.26 | 0.79 |
| Kucukhuseyin O [R112] | 2013 | M/F | Other ethnicity | Case/Control | PCR-RFLP | 194/138 | 103 | 68 | 23 |  | 62 | 61 | 15 | 0.33 | 1.00 |
| Davis LA [R113] | 2013 | M/F | Caucasian | Nested Case/Control | DNA sequencing | 97/948 | 53 | 42 | 2 |  | 471 | 374 | 103 | 0.31 | 0.03 |
| Saffari B [R114] | 2013 | M/F | Caucasian | Case/Control | PCR-RFLP | 457/371 | 242 | 170 | 45 |  | 182 | 160 | 29 | 0.29 | 0.45 |
| Senemar S [R115] | 2013 | M/F | Caucasian | Case/Control | PCR-RFLP | 231/300 | 118 | 88 | 25 |  | 174 | 102 | 24 | 0.25 | 0.11 |
| Tang O [R116] | 2014 | M/F | Asian | Case/Control | DNA sequencing | 50/25 | 15 | 27 | 8 |  | 14 | 9 | 2 | 0.26 | 0.75 |
| Yu X [R117] | 2014 | M/F | Asian | Case/Control | DNA sequencing | 1133/1106 | 401 | 523 | 209 |  | 422 | 542 | 142 | 0.37 | 0.12 |
| Chen W [R118] | 2014 | M/F | Asian | Case/Control | DNA sequencing | 424/476 | 98 | 209 | 117 |  | 141 | 221 | 114 | 0.47 | 0.14 |
| Ramkaran P [R119] | 2015 | M | Asian | Case/Control | PCR-RFLP | 106/100 | 79 | 25 | 2 |  | 86 | 14 | 0 | 0.07 | 0.45 |
| Tanguturi PR [R120] | 2015 | M/F | Asian | Case/Control | PCR-RFLP | 202/210 | 96 | 85 | 21 |  | 103 | 88 | 19 | 0.30 | 0.97 |
| Heidari MM [R121] | 2015 | M/F | Caucasian | Case/Control | PCR-RFLP | 108/90 | 54 | 36 | 18 |  | 61 | 27 | 2 | 0.17 | 0.62 |
| Lin X [R122] | 2016 | M/F | Asian | Case/Control | PCR-RFLP | 105/105 | 45 | 42 | 18 |  | 63 | 35 | 7 | 0.23 | 0.48 |
| Bickel C [R123] | 2017 | M/F | Caucasian | Nested Case/Control | Real-time PCR assay | 1030/320 | 427 | 484 | 119 |  | 143 | 143 | 34 | 0.33 | 0.84 |

*MTHFR*: 5,10-methylenetetrahydrofolate reductase gene; M: male; F: female; CAD: coronary artery disease; PCR-RFLP: polymerase chain reaction-restricted fragment length polymorphism.

**Table S2. Characteristics of the individual studies included in the meta-analysis between the *MTHFR* rs1801133 polymorphism and plasma lipid levels**

| **First author, reference** | **year** | **Ethnicity** | **Gender** | **Genotyping method** | **Study population** | **Outcomes** |
| --- | --- | --- | --- | --- | --- | --- |
| Mazza A [R124] | 2000 | Caucasian | M/F | PCR-RFLP | Non-insulin-dependent diabetes mellitus patients | TG, TC, HDL-C |
| Passaro A [R125] | 2001 | Caucasian | F | PCR-RFLP | Healthy subjects | TG, TC, LDL-C, HDL-C |
| Vulapalli R [R126] | 2001 | Caucasian | M/F | PCR-RFLP | CAD patients | TG, TC, LDL-C, HDL-C |
| Hu S [R127] | 2001 | Asian | M/F | PCR-RFLP | Diabetes mellitus patients and control subjects | TG, TC, LDL-C, HDL-C |
| Kawamoto R [R128] | 2001 | Asian | M/F | PCR-RFLP | CAD high-risk subjects | TG, TC, HDL-C |
| Mao YM [R129] | 2002 | Asian | M/F | PCR-RFLP | CAD patients and control subjects | TG, TC, HDL-C |
| Butler R [R130] | 2002 | Caucasian | M | Not clear | Healthy subjects | TC, HDL-C |
| Vasisht S1 [R58] | 2002 | Caucasian | M/F | PCR-RFLP | CAD patients | TG, TC, LDL-C, HDL-C |
| Vasisht S2 [R58] | 2002 | Caucasian | M/F | PCR-RFLP | Healthy subjects | TG, TC, LDL-C, HDL-C |
| Frederiksen J [R74] | 2004 | Caucasian | M/F | PCR-RFLP | Healthy subjects | TG, TC, HDL-C |
| Tutuncu NB [R131] | 2005 | Other ethnicity | M/F | PCR-RFLP | T2DM patients | LDL-C, HDL-C |
| Pollex RL [R132] | 2005 | Caucasian | M/F | PCR-RFLP | T2DM patients | TG, TC, LDL-C, HDL-C |
| Winkelmayer WC [R133] | 2005 | Caucasian | M/F | PCR-RFLP | Kidney transplant recipients | TG, TC |
| Kerkeni M [R84] | 2006 | African | M/F | PCR-RFLP | CAD patients | TG, TC, LDL-C, HDL-C |
| Koubaa N [R134] | 2007 | African | M/F | PCR-RFLP | T2DM patients | TC, LDL-C, HDL-C |
| Pereira AC [R135] | 2007 | Other ethnicity | M/F | PCR-RFLP | CAD patients | TG, TC, LDL-C, HDL-C |
| Zee RY [R136] | 2007 | Caucasian | F | Real-time PCR assay | Healthy subjects | TC, HDL-C |
| Yao H [R137] | 2007 | Asian | M | Real-time PCR assay | Hyperuricemia patients | TG, TC, LDL-C, HDL-C |
| Maeda M [R138] | 2008 | Asian | M/F | PCR-RFLP | T2DM patients | TG, TC, LDL-C, HDL-C |
| Alam MA [R91] | 2008 | Asian | M/F | PCR-RFLP | CAD patients | TG, TC, LDL-C, HDL-C |
| Huang L1 [R139] | 2008 | Asian | M/F | PCR-RFLP | Hyperlipidemia patients | TG, TC, LDL-C, HDL-C |
| Huang L2 [R139] | 2008 | Asian | M/F | PCR-RFLP | Control subjects | TG, TC, LDL-C, HDL-C |
| Collings A1 [R140] | 2008 | Caucasian | M | Real-time PCR assay | Healthy subjects | TG, TC, LDL-C, HDL-C |
| Collings A2 [R140] | 2008 | Caucasian | F | Real-time PCR assay | Healthy subjects | TG, TC, LDL-C, HDL-C |
| Maitland-van der Zee AH [R141] | 2008 | Other ethnicity | M/F | Real-time PCR assay | Hypertension patients | TC, LDL-C, HDL-C |
| Karadeniz M [R142] | 2010 | Other ethnicity | F | Real-time PCR assay | Polycystic ovary syndrome patients | TG, TC, LDL-C, HDL-C |
| Chen AR [R143] | 2010 | Asian | M/F | PCR-RFLP | T2DM and metabolic syndrome patients | TG, HDL-C |
| Iemitsu M1 [R144] | 2010 | Asian | M/F | Real-time PCR assay | Healthy subjects | TG, TC, HDL-C |
| Iemitsu M2 [R144] | 2010 | Asian | M/F | Real-time PCR assay | Healthy subjects | TG, TC, HDL-C |
| Zhang L1 [R145] | 2010 | Asian | M | PCR-RFLP | Healthy subjects | TC, LDL-C, HDL-C |
| Zhang L2 [R145] | 2010 | Asian | F | PCR-RFLP | Healthy subjects | TC, LDL-C, HDL-C |
| Zhang L3 [R145] | 2010 | Asian | M | PCR-RFLP | Healthy subjects | TC, LDL-C, HDL-C |
| Zhang L4 [R145] | 2010 | Asian | F | PCR-RFLP | Healthy subjects | TC, LDL-C, HDL-C |
| Lin L [R146] | 2010 | Asian | M/F | PCR-RFLP | Diabetic nephropathy patients | TG, TC, LDL-C, HDL-C |
| Miranda-Vilela AL [R147] | 2011 | Other ethnicity | M/F | PCR-RFLP | Healthy subjects | TG, TC, LDL-C, HDL-C |
| Siqueira ER [R148] | 2011 | Other ethnicity | M/F | PCR-RFLP | Chronic hepatitis C infection patients | TG, TC, LDL-C, HDL-C |
| Taguchi T [R149] | 2012 | Asian | F | PCR-RFLP | Healthy subjects | TG, TC, LDL-C, HDL-C |
| Yang Q [R150] | 2012 | Other ethnicity | M/F | Real-time PCR assay | Healthy subjects | TC |
| Qin X [R151] | 2012 | Asian | M/F | PCR-RFLP | Hypertension patients | TG, TC, HDL-C |
| Trifonova EA1 [R111] | 2012 | Caucasian | M/F | PCR-RFLP | CAD patients | TG, TC, HDL-C |
| Trifonova EA2 [R111] | 2012 | Caucasian | M/F | PCR-RFLP | Control subjects | TG, TC, HDL-C |
| Jain M [R152] | 2012 | Asian | F | PCR-RFLP | Polycystic ovary syndrome patients | TG, TC, LDL-C, HDL-C |
| Ford AH [R153] | 2012 | Caucasian | M | PCR-RFLP | Cognitive impairment patients and controls subjects | TG, TC, LDL-C |
| Devlin AM [R154] | 2012 | Caucasian | M/F | Real-time PCR assay | Psychotics patients | TG, TC, LDL-C, HDL-C |
| Chmurzynska A [R155] | 2013 | Caucasian | F | PCR-RFLP | Healthy subjects | TG, TC, LDL-C, HDL-C |
| Senemar S [R115] | 2013 | Caucasian | M/F | PCR-RFLP | CAD patients | TG, TC, LDL-C, HDL-C |
| Lambrinoudaki I [R156] | 2013 | Caucasian | F | Real-time PCR assay | Healthy subjects | TG, TC, LDL-C, HDL-C |
| Saffari B [R114] | 2013 | Caucasian | M/F | PCR-RFLP | CAD patients | TG, TC, LDL-C, HDL-C |
| Yin RX1 [R157] | 2013 | Asian | M/F | PCR-RFLP | Healthy subjects | TG, TC, LDL-C, HDL-C |
| Yin RX2 [R157] | 2013 | Asian | M/F | PCR-RFLP | Healthy subjects | TG, TC, LDL-C, HDL-C |
| Bahadir A1 [R158] | 2013 | Other ethnicity | M/F | PCR-RFLP | Migraine patients | TG, TC, LDL-C, HDL-C |
| Bahadir A2 [R158] | 2013 | Other ethnicity | M/F | PCR-RFLP | Control subjects | TG, TC, LDL-C, HDL-C |
| Yigit S [R159] | 2013 | Other ethnicity | M/F | PCR-RFLP | Diabetic peripheral neuropathy patients | TG, TC, LDL-C, HDL-C |
| Kucukhuseyin O1 [R112] | 2013 | Other ethnicity | M/F | PCR-RFLP | DM (-) CAD patients | TG, TC, LDL-C, HDL-C |
| Kucukhuseyin O2 [R112] | 2013 | Other ethnicity | M/F | PCR-RFLP | DM (+) CAD patients | TG, TC, LDL-C, HDL-C |
| Chen NY1 [R160] | 2014 | Asian | M | PCR-RFLP | Healthy subjects | TC, LDL-C, HDL-C |
| Chen NY2 [R160] | 2014 | Asian | F | PCR-RFLP | Healthy subjects | TC, LDL-C, HDL-C |
| Chen NY3 [R160] | 2014 | Asian | M | PCR-RFLP | Healthy subjects | TC, LDL-C, HDL-C |
| Chen NY4 [R160] | 2014 | Asian | F | PCR-RFLP | Healthy subjects | TC, LDL-C, HDL-C |
| Bahadır A [R161] | 2014 | Other ethnicity | M/F | PCR-RFLP | Severe CVD and T2DM patients | TC |
| Asefi M1 [R162] | 2014 | Caucasian | M/F | DNA sequencing | Psoriasis patients | TG, TC, LDL-C, HDL-C |
| Asefi M2 [R162] | 2014 | Caucasian | M/F | DNA sequencing | Healthy subjects | TG, TC, LDL-C, HDL-C |
| Jiang S [R163] | 2014 | Asian | M/F | Real-time PCR assay | Hypertension patients | TG, TC, LDL-C, HDL-C |
| LV CF [R164] | 2014 | Asian | M/F | PCR-RFLP | Hypertension patients | TC, LDL-C |
| Liang RL [R165] | 2014 | Asian | M/F | PCR-RFLP | Hyperlipidemia patients and controls subjects | TG, TC, LDL-C, HDL-C |
| Husemoen LL [R166] | 2014 | Caucasian | M/F | DNA sequencing | Healthy subjects | TG, HDL-C |
| Li WX [R167] | 2015 | Asian | M/F | PCR-RFLP | Hypertension patients | TG, TC, LDL-C, HDL-C |
| Tetik Vardarlı A [R168] | 2015 | Other ethnicity | M/F | Real-time PCR assay | Acromegaly patients | TG, TC, LDL-C, HDL-C |
| Ramkaran P [R119] | 2015 | Asian | M | PCR-RFLP | CAD patients | TG, TC, LDL-C, HDL-C |
| Mohammadzadeh G [R169] | 2015 | Caucasian | M/F | PCR-RFLP | Breast cancer patients and controls subjects | TG, TC, LDL-C, HDL-C |
| Chen SF [R170] | 2015 | Asian | M/F | DNA sequencing | CAD patients | TG, TC, LDL-C, HDL-C |
| Morais CC [R171] | 2015 | Other ethnicity | M/F | Real-time PCR assay | Obesity/Overweight patients | TG, TC, LDL-C, HDL-C |
| Zhi X1 [R172] | 2016 | Asian | M | Real-time PCR assay | Healthy subjects | TC, LDL-C, HDL-C |
| Zhi X2 [R172] | 2016 | Asian | M | Real-time PCR assay | Healthy subjects | TC, LDL-C, HDL-C |
| Zhi X3 [R172] | 2016 | Asian | F | Real-time PCR assay | Healthy subjects | TC, LDL-C, HDL-C |
| Zhi X4 [R172] | 2016 | Asian | F | Real-time PCR assay | Healthy subjects | TC, LDL-C, HDL-C |
| Ghogomu SM [R173] | 2016 | African | M/F | PCR-RFLP | Hypertension patients and controls subjects | TG, TC, LDL-C |
| El Hajj Chehadeh SW [R174] | 2016 | Caucasian | M/F | Real-time PCR assay | T2DM patients | TG, TC, LDL-C, HDL-C |
| Abd-Elmawla MA [R175] | 2016 | Caucasian | M/F | PCR-RFLP | β-thalasemia major (β-TM) patients | LDL-C, HDL-C |
| Rashed L [R176] | 2017 | Caucasian | M/F | PCR-RFLP | Cutaneous lichen planus or oral lichen planus patients | TG, TC, HDL-C |
| Fan GJ [R177] | 2017 | Asian | M/F | Real-time PCR assay | Hypertension patients | TG, TC, LDL-C, HDL-C |
| Shang GY [R178] | 2017 | Asian | M/F | PCR-based gene chip | Diabetes mellitus patients and controls subjects | TG, TC, LDL-C, HDL-C |
| Abd El-Aziz TA [R179] | 2017 | Caucasian | F | PCR-RFLP | Rheumatoid arthritis patients | TG, TC |

*MTHFR*: 5,10-methylenetetrahydrofolate reductase gene; M: male; F: female; CAD: coronary artery disease; T2DM: type 2 diabetes mellitus; TG: triglycerides; TC: total cholesterol; LDL-C: low-density lipoprotein cholesterol; HDL-C: high-density lipoprotein cholesterol; PCR-RFLP: polymerase chain reaction-restricted fragment length polymorphism.

**Table S3. Plasma lipid levels according to the genotypes of the *MTHFR* rs1801133 polymorphism**

| **First author, reference** | **Number** | |  | **TG, mmol/L** | |  | **TC, mmol/L** | |  | **LDL-C, mmol/L** | |  | **HDL-C, mmol/L** | |
| --- | --- | --- | --- | --- | --- | --- | --- | --- | --- | --- | --- | --- | --- | --- |
|  | **CC** | **CT+TT** |  | **CC** | **CT+TT** |  | **CC** | **CT+TT** |  | **CC** | **CT+TT** |  | **CC** | **CT+TT** |
| Mazza A [R124] | 47 | 83 |  | 1.60 ± 0.96 | 1.75± 1.04 |  | 5.00 ± 1.30 | 5.18 ± 1.05 |  | - | - |  | 1.20 ± 0.46 | 1.26 ± 0.44 |
| Passaro A [R125] | 28 | 92 |  | 1.48±0.32 | 1.53±0.24 |  | 5.43±0.39 | 5.63±0.33 |  | 3.31±0.52 | 3.37±0.49 |  | 1.37±0.34 | 1.25±0.29 |
| Vulapalli R [R126] | 675 | 343 |  | 2.22 ± 1.25 | 2.34 ± 1.47 |  | 5.09 ± 1.11 | 5.1 ± 1.17 |  | 3.13 ± 1.01 | 3.06± 0.98 |  | 1.01± 0.28 | 1.02 ± 0.33 |
| Hu S [R127] | 88 | 80 |  | 5.29± 1.21 | 5.27± 1.12 |  | 1.57± 1.08 | 1.58± 1.29 |  | 3.19± 0.61 | 3.09± 0.57 |  | 1.14± 0.35 | 1.23± 0.26 |
| Kawamoto R [R128] | 136 | 190 |  | 1.02±0.51 | 1.05±0.61 |  | 4.76±0.96 | 4.58±1.04 |  | - | - |  | 1.34±0.49 | 1.28±0.48 |
| Mao YM [R129] | 80 | 354 |  | 1.33 ± 0.66 | 1.52± 1.05 |  | 5.01 ± 0.73 | 5.23±0.89 |  | - | - |  | 1.24 ± 0.24 | 1.21 ± 0.24 |
| Butler R [R130] | 36 | 32 |  | - | - |  | 3.9 ±0.8 | 4.1± 1.0 |  | - | - |  | 1.25 ±0.30 | 1.18 ±0.17 |
| Vasisht S1 [R58] | 98 | 43 |  | 2±1.21 | 1.72±0.72 |  | 5.28±1.5 | 5.11±1.23 |  | 3±1.03 | 2.96±1.01 |  | 1.06±0.14 | 1.14±0.27 |
| Vasisht S2 [R58] | 39 | 16 |  | 2±1.49 | 1.8±0.68 |  | 4.99±1.16 | 5.8±0.4 |  | 2.79±1.09 | 2.38±1.02 |  | 1.08± 0.18 | 1.04± 0.1 |
| Frederiksen J [R74] | 4429 | 4809 |  | 1.7 ± 1.33 | 1.7 ± 1.24 |  | 5.8 ± 1.33 | 5.8 ±1.24 |  | - | - |  | 1.3 ±0.67 | 1.3 ± 0.58 |
| Tutuncu NB [R131] | 42 | 52 |  | - | - |  | - | - |  | 3.57± 0.78 | 3.62 ± 0.9 |  | 1.19 ± 0.41 | 1.14 ± 0.35 |
| Pollex RL [R132] | 108 | 30 |  | 2.20 ± 2.78 | 1.66 ± 0.60 |  | 4.91 ± 1.07 | 4.67 ± 0.82 |  | 2.75 ± 0.71 | 2.70 ± 0.67 |  | 1.21 ± 0.29 | 1.21 ± 0.29 |
| Winkelmayer WC [R133] | 334 | 376 |  | 2.32±2.16 | 2.08±1.21 |  | 6.08± 1.58 | 6.03 ±1.31 |  | - | - |  | - | - |
| Kerkeni M [R84] | 49 | 51 |  | 1.42 ± 1.10 | 1.67 ± 0.80 |  | 4.93 ± 0.99 | 5.25± 0.84 |  | 4.08 ± 1.04 | 4.11 ± 0.99 |  | 0.71 ± 0.12 | 0.73 ± 0.15 |
| Koubaa N [R134] | 41 | 45 |  | - | - |  | 4.5±1.3 | 4.4±1.2 |  | 2.9±1.4 | 2.4±1.07 |  | 1.01±0.29 | 1.05±0.27 |
| Pereira AC [R135] | 250 | 308 |  | 2.18±1.3 | 2.17±1.32 |  | 5.73±1.24 | 5.8±1.24 |  | 3.77±1.17 | 3.83±1.1 |  | 0.96±0.27 | 0.97±0.28 |
| Zee RY [R136] | 11229 | 13739 |  | - | - |  | 5.46± 1.06 | 5.48± 1.08 |  | - | - |  | 1.39 ±0.39 | 1.39 ±0.39 |
| Yao H [R137] | 19 | 73 |  | 2.13±1.39 | 2.45±1.78 |  | 5.27±1.00 | 5.36±0.92 |  | 2.89±0.74 | 2.85±0.74 |  | 1.70±0.28 | 1.67±0.28 |
| Maeda M [R138] | 74 | 116 |  | 1.8±2.0 | 1.74±1.38 |  | 5.1±1.0 | 5.36±0.95 |  | 2.9±1.0 | 3.11±0.81 |  | 1.4±0.4 | 1.42±0.4 |
| Alam MA [R91] | 57 | 27 |  | 1.72± 0.65 | 1.66 ±0.86 |  | 4.72 ± 1.16 | 4.97± 1.18 |  | 2.94 ± 0.96 | 3.14 ± 0.9 |  | 1.03 ± 0.15 | 1.03 ± 0.11 |
| Huang L1 [R139] | 40 | 152 |  | 1.87±0.95 | 2.24±1.75 |  | 5.51±0.92 | 5.61±1.00 |  | 3.00±0.84 | 3.02±0.89 |  | 1.35±0.35 | 1.36±0.38 |
| Huang L2 [R139] | 51 | 137 |  | 1.01±0.37 | 0.91±0.32 |  | 4.32±0.57 | 4.22±0.65 |  | 2.41±0.51 | 2.23±0.57 |  | 1.47±0.23 | 1.35±0.27 |
| Collings A1 [R140] | 416 | 304 |  | 1.49 ±0.93 | 1.49± 0.86 |  | 5.27 ±1.02 | 5.18 ±0.99 |  | 3.42 ±0.96 | 3.38±0.87 |  | 1.18± 0.29 | 1.15 ±0.26 |
| Collings A2 [R140] | 419 | 301 |  | 1.18± 0.82 | 1.17 ±0.58 |  | 5.11± 0.95 | 5.06± 0.93 |  | 3.17 ±0.77 | 3.14 ±0.79 |  | 1.40 ±0.30 | 1.38± 0.29 |
| Maitland-van der Zee AH [R141] | 5310 | 4306 |  | - | - |  | 5.79± 0.69 | 5.77± 0.69 |  | 3.77 ±0.55 | 3.75 ±0.56 |  | 1.25 ±0.36 | 1.19±0.33 |
| Karadeniz M [R142] | 15 | 71 |  | 1.363 ± 1.01 | 1.58 ± 0.87 |  | 4.65 ± 0.89 | 5.03± 1.1 |  | 2.78 ± 0.65 | 3.07 ± 0.88 |  | 1.48 ± 0.25 | 1.43 ± 0.4 |
| Chen AR [R143] | 34 | 84 |  | 1.74±1.07 | 2.58±1.82 |  | - | - |  | - | - |  | 1.32±0.32 | 1.27±0.36 |
| Iemitsu M1 [R144] | 134 | 244 |  | 0.85±0.39 | 0.8±0.31 |  | 4.91± 0.9 | 4.91 ± 1.08 |  | - | - |  | 1.68 ±0.3 | 1.64 ±0.33 |
| Iemitsu M2 [R144] | 134 | 251 |  | 0.77±0.39 | 0.75 ±0.32 |  | 4.89± 0.9 | 4.96± 1.06 |  | - | - |  | 1.73±0.3 | 1.81 ± 0.37 |
| Zhang L1 [R145] | 228 | 153 |  | - | - |  | 4.20 ± 0.90 | 4.37 ± 1.21 |  | 2.38 ± 0.75 | 2.57 ± 0.96 |  | 1.64 ± 0.45 | 1.69 ± 0.49 |
| Zhang L2 [R145] | 230 | 169 |  | - | - |  | 4.21 ± 0.80 | 4.31 ± 0.82 |  | 2.51 ± 0.63 | 2.59± 0.65 |  | 1.61 ± 0.35 | 1.67 ± 0.39 |
| Zhang L3 [R145] | 107 | 235 |  | - | - |  | 4.58 ± 0.86 | 4.78 ± 0.96 |  | 2.45 ± 0.69 | 2.64 ± 0.73 |  | 1.88 ± 0.57 | 1.86 ± 0.5 |
| Zhang L4 [R145] | 117 | 227 |  | - | - |  | 4.55 ± 0.76 | 4.81 ± 0.97 |  | 2.50 ± 0.61 | 2.65 ± 0.75 |  | 1.93 ± 0.46 | 1.98 ± 0.45 |
| Lin L [R146] | 19 | 44 |  | 1. 62± 0. 60 | 2.07± 0.95 |  | 5. 28± 1. 35 | 5.29± 1.19 |  | 2. 81± 1. 11 | 3.28± 0.68 |  | 1. 36± 0. 32 | 1.34± 0.28 |
| Miranda-Vilela AL [R147] | 59 | 66 |  | 1.25 ± 0.71 | 1.3 ± 0.7 |  | 4.84 ± 1.03 | 4.86± 1.02 |  | 2.92 ± 0.95 | 2.82 ± 0.76 |  | 1.37 ± 0.32 | 1.43 ± 0.38 |
| Siqueira ER [R148] | 73 | 65 |  | 1.12 ± 0.52 | 1.17 ± 0.46 |  | 4.02± 1.13 | 3.81 ± 0.87 |  | 2.25± 1.03 | 2.06 ± 0.79 |  | 1.27 ± 0.36 | 1.23 ± 0.37 |
| Taguchi T [R149] | 72 | 120 |  | 0.95± 0.59 | 0.81 ± 0.39 |  | 4.47 ± 0.79 | 4.48 ± 0.75 |  | 2.39 ± 0.61 | 2.46 ± 0.64 |  | 1.68± 0.35 | 1.64 ± 0.29 |
| Yang Q [R150] | 2984 | 2941 |  | - | - |  | 5.11± 1.99 | 5.14 ±1.58 |  | - | - |  | - | - |
| Qin X [R151] | 114 | 331 |  | 1.7±1.0 | 1.77±1.31 |  | 5.0±1.4 | 4.97±1.0 |  | - | - |  | 1.3±0.4 | 1.3±0.4 |
| Trifonova EA1 [R111] | 52 | 48 |  | 1.61 ± 0.72 | 1.46 ± 0.75 |  | 5.66 ± 1.15 | 5.31 ± 1.03 |  | - | - |  | 0.97 ± 0.29 | 0.95 ± 0.35 |
| Trifonova EA2 [R111] | 55 | 45 |  | 1.42 ± 1.26 | 1.2 ± 0.7 |  | 6.03 ± 1.48 | 5.66 ± 1.36 |  | - | - |  | 1.33 ± 0.3 | 1.29 ± 0.3 |
| Jain M [R152] | 76 | 16 |  | 0.93±0.24 | 1.08±0.42 |  | 5.26±0.42 | 5.57±0.66 |  | 1.82±0.55 | 2.14±0.99 |  | 1.27±0.18 | 1.2±0.27 |
| Ford AH [R153] | 722 | 878 |  | 1.2 ±1.6 | 1.2 ±1.6 |  | 4.9 ±1.0 | 4.9 ±0.9 |  | 2.9 ±0.8 | 2.9± 0.8 |  | - | - |
| Devlin AM [R154] | 54 | 45 |  | 1.10 ±0.71 | 1.09± 0.57 |  | 4.58± 1.03 | 4.62 ±1.06 |  | 2.71± 0.92 | 2.91± 1.00 |  | 1.35 ±0.36 | 1.27 ±0.42 |
| Chmurzynska A [R155] | 52 | 70 |  | 1.22 ± 0.55 | 1.43± 0.59 |  | 6.16 ± 0.84 | 6.18 ±1.21 |  | 3.67 ±0.73 | 3.78 ± 1 |  | 1.93 ± 0.47 | 1.76± 0.43 |
| Senemar S [R115] | 118 | 113 |  | 1.74 ± 0.67 | 1.75 ± 0.66 |  | 4.05 ± 1.53 | 4.11± 1.62 |  | 2.97 ± 1.42 | 3.1± 1.56 |  | 0.94 ± 0.24 | 0.95 ± 0.23 |
| Lambrinoudaki I [R156] | 33 | 64 |  | 1.15 ± 0.65 | 1.07±0.46 |  | 5.97±1.13 | 5.82±0.99 |  | 3.8±1.09 | 3.82±1.05 |  | 1.6± 0.32 | 1.55±0.34 |
| Saffari B [R114] | 242 | 215 |  | 1.64±0.76 | 1.62±0.78 |  | 3.89±1.76 | 3.92±1.95 |  | 3.19±1.49 | 3.11±1.57 |  | 1.2±0.96 | 1.2±1.04 |
| Yin RX1 [R157] | 471 | 507 |  | 1.13 ± 0.89 | 1.17 ± 0.9 |  | 4.20 ± 0.81 | 4.49± 0.98 |  | 2.39 ± 0.65 | 2.54 ± 0.74 |  | 1.71 ± 0.46 | 1.83 ± 0.49 |
| Yin RX2 [R157] | 354 | 397 |  | 1.63 ± 1.30 | 1.86 ±2.29 |  | 4.72 ± 0.88 | 4.97± 1.2 |  | 2.77 ± 0.75 | 2.83± 0.87 |  | 1.66 ± 0.38 | 1.80 ± 0.43 |
| Bahadir A1 [R158] | 50 | 100 |  | 1.41 ± 1.06 | 1.53 ± 1.02 |  | 4.96 ± 0.9 | 4.86±1.03 |  | 2.96 ± 0.8 | 2.8 ± 0.93 |  | 1.52± 0.78 | 1.38± 0.33 |
| Bahadir A2 [R158] | 96 | 11 |  | 1.43 ± 0.93 | 1.76 ± 0.89 |  | 4.46 ± 1.03 | 5.01± 1.36 |  | 2.64± 0.78 | 3.2 ± 0.95 |  | 1.32 ± 0.51 | 1.19 ± 0.24 |
| Yigit S [R159] | 123 | 107 |  | 1.81±0.62 | 1.96±1.15 |  | 4.8±1.18 | 4.9±1.57 |  | 3.32±0.83 | 3.38±0.85 |  | 1.08±0.26 | 1.07±0.25 |
| Kucukhuseyin O1 [R112] | 63 | 49 |  | 1.59 ± 0.82 | 1.68 ± 0.64 |  | 5.54 ± 1.59 | 5.64 ± 1.09 |  | 3.46 ± 1.22 | 3.71 ± 0.94 |  | 1.03 ± 0.17 | 1.03 ± 0.21 |
| Kucukhuseyin O2 [R112] | 40 | 42 |  | 1.92 ± 0.88 | 1.67 ± 1.27 |  | 5.19 ± 1.40 | 4.87 ± 1.31 |  | 3.25 ± 1.08 | 2.90 ± 0.99 |  | 0.96 ± 0.25 | 1.00 ± 0.25 |
| Chen NY1 [R160] | 83 | 44 |  | - | - |  | 5.00 ± 1.11 | 4.68 ± 1.09 |  | 2.96 ± 0.95 | 2.69 ± 0.92 |  | 1.57 ± 0.41 | 1.54 ± 0.44 |
| Chen NY2 [R160] | 249 | 140 |  | - | - |  | 5.15 ± 0.96 | 5.37 ± 1.05 |  | 3.06 ± 0.84 | 3.2± 0.83 |  | 1.60 ± 0.35 | 1.62 ± 0.39 |
| Chen NY3 [R160] | 95 | 47 |  | - | - |  | 4.99 ± 0.99 | 4.85 ± 0.84 |  | 2.75 ± 0.96 | 2.69 ± 0.69 |  | 1.63 ± 0.41 | 1.67 ± 0.46 |
| Chen NY4 [R160] | 273 | 78 |  | - | - |  | 4.96 ± 0.94 | 5.05 ± 1.07 |  | 2.69 ± 0.88 | 2.88± 0.9 |  | 1.76 ± 0.40 | 1.68 ± 0.39 |
| Bahadır A [R161] | 31 | 76 |  | - | - |  | 4.73±0.87 | 4.8±1.12 |  | - | - |  | - | - |
| Asefi M1 [R162] | 50 | 50 |  | 1.41 ± 0.6 | 1.38 ± 0.9 |  | 4.42± 1.04 | 4.27 ± 1.17 |  | 2.92 ± 0.96 | 2.9 ± 0.97 |  | 1.08 ± 0.38 | 1.16 ± 0.4 |
| Asefi M2 [R162] | 64 | 36 |  | 1.46 ± 0.79 | 1.36± 0.58 |  | 4.16± 0.95 | 4.22 ± 0.92 |  | 2.69 ± 0.7 | 2.84 ± 0.76 |  | 1.16± 0.36 | 1.19 ± 0.35 |
| Jiang S [R163] | 133 | 207 |  | 1.29 ± 0.65 | 1.27 ± 0.67 |  | 4.57 ± 0.70 | 4.71± 0.82 |  | 2.34 ± 0.59 | 2.48 ± 0.78 |  | 1.64 ± 0.52 | 1.65± 0.54 |
| LV CF [R164] | 57 | 93 |  | - | - |  | 5.06±0.98 | 5.14±1.03 |  | 3.11±0.82 | 3.2±0.88 |  | - | - |
| Liang RL [R165] | 976 | 615 |  | 1.46 ±1.12 | 1.46±1.05 |  | 4.59 ± 1.17 | 4.58± 1.25 |  | 2.74±0.88 | 2.76±0.94 |  | 1.20±0.31 | 1.21±0.33 |
| Husemoen LL [R166] | 6037 | 6391 |  | 1.37± 1.31 | 1.40± 1.1 |  | - | - |  | - | - |  | 1.49± 0.42 | 1.47± 0.42 |
| Li WX [R167] | 108 | 317 |  | 1.75 ± 1.06 | 1.73 ± 1.3 |  | 5.01 ± 1.42 | 5.0±0.99 |  | 2.87 ± 0.83 | 2.93 ± 0.78 |  | 1.33 ± 0.36 | 1.3 ± 0.36 |
| Tetik Vardarlı A [R168] | 36 | 55 |  | 1.47 ± 0.87 | 1.41±0.77 |  | 5.16 ±0.98 | 4.83 ± 1.03 |  | 3.03 ± 0.97 | 2.84 ± 0.92 |  | 1.42 ± 0.38 | 1.3± 0.27 |
| Ramkaran P [R119] | 79 | 27 |  | 2.45 ± 1.6 | 2.13 ± 1.35 |  | 5.43 ± 1.87 | 5.10 ± 1.66 |  | 3.45 ± 1.78 | 3.23 ± 1.61 |  | 0.93 ± 0.27 | 0.90 ± 0.31 |
| Mohammadzadeh G [R169] | 125 | 108 |  | 1.43±0.52 | 1.34±0.55 |  | 5.34±1.25 | 4.94±1.14 |  | 2.43±0.39 | 2.45±0.42 |  | 1.3±0.31 | 1.32±0.27 |
| Chen SF [R170] | 82 | 204 |  | 1.15±0.46 | 1.51±0.62 |  | 4.38±0.87 | 4.61±0.68 |  | 2.76±0.81 | 2.88±0.61 |  | 1.21±0.29 | 1.14±0.22 |
| Morais CC [R171] | 69 | 46 |  | 0.99± 0.48 | 1.16± 0.72 |  | 4 ± 0.72 | 4.05 ± 0.63 |  | 1.21 ± 0.29 | 1.18± 0.33 |  | 2.34 ± 0.57 | 2.33± 0.5 |
| Zhi X1 [R172] | 94 | 321 |  | - | - |  | 4.83 ± 1.12 | 4.84± 1.05 |  | 2.65 ± 1.01 | 2.65± 0.93 |  | 1.24 ± 0.35 | 1.26 ± 0.30 |
| Zhi X2 [R172] | 226 | 848 |  | - | - |  | 4.99 ± 0.87 | 5.1 ± 0.95 |  | 2.76 ± 0.89 | 2.87 ± 1 |  | 1.14 ± 0.30 | 1.12 ± 0.27 |
| Zhi X3 [R172] | 104 | 366 |  | - | - |  | 4.82 ± 1.03 | 4.84 ± 0.94 |  | 2.66 ± 0.93 | 2.67 ± 0.95 |  | 1.39 ± 0.38 | 1.38 ± 0.37 |
| Zhi X4 [R172] | 61 | 219 |  | - | - |  | 5.13 ± 0.98 | 5.0 ± 1.03 |  | 2.90 ± 0.87 | 2.75 ± 0.95 |  | 1.28 ± 0.36 | 1.21± 0.31 |
| Ghogomu SM [R173] | 51 | 40 |  | 1.27± 0.55 | 1.33 ± 0.35 |  | 5.43 ± 1.08 | 4.2 ± 0.85 |  | 2.47± 0.74 | 5.3 ± 0.7 |  | - | - |
| El Hajj Chehadeh SW [R174] | 155 | 54 |  | 1.35 ± 0.65 | 1.58 ± 0.92 |  | 4.00 ± 0.92 | 4.28 ± 1.3 |  | 2.15 ± 0.81 | 2.5 ± 1.13 |  | 1.21 ± 0.31 | 1.18± 0.37 |
| Abd-Elmawla MA [R175] | 48 | 18 |  | - | - |  | - | - |  | 3.17 ± 1.62 | 3.39 ± 1.49 |  | 1.2 ± 0.37 | 0.84± 0.23 |
| Rashed L [R176] | 52 | 58 |  | 1.09±0.18 | 1.05±0.16 |  | 5.93±0.67 | 5.87±0.88 |  | - | - |  | 1.05±0.25 | 1.02±0.28 |
| Fan GJ [R177] | 46 | 266 |  | 1.50±0.92 | 1.75±1.44 |  | 4.70±0.99 | 4.83±1.07 |  | 2.88±0.87 | 3.03±0.82 |  | 1.38±0.31 | 1.34±0.35 |
| Shang GY [R178] | 314 | 189 |  | 1.49 ± 0.98 | 1.46 ± 1.02 |  | 4.30 ± 1.01 | 4.39 ± 1.02 |  | 3.01 ± 0.77 | 3.03 ± 0.73 |  | 1.28 ± 0.38 | 1.32 ± 0.38 |
| Abd El-Aziz TA [R179] | 79 | 81 |  | 0.82± 0.26 | 0.95 ± 0.35 |  | 4.17± 0.38 | 4.33± 0.46 |  | - | - |  | - | - |

*MTHFR*: 5,10-methylenetetrahydrofolate reductase gene; TG: triglycerides; TC: total cholesterol; LDL-C: low-density lipoprotein cholesterol; HDL-C: high-density lipoprotein cholesterol.
